# Analyzing DNA Replication Fork Stability and Collapse Using Chromatin Fiber Analysis and the R-ODD-BLOBS Program

**DOI:** 10.34133/csbj.0140

**Published:** 2026-07-22

**Authors:** Kerenza Cheng, Kazeera Aliar, Roozbeh Manshaei, Susan L. Forsburg, Ali Mazalek, Sarah A. Sabatinos

**Affiliations:** ^1^ Molecular Science Graduate Program, Yeates School of Graduate and Postdoctoral Studies, Toronto Metropolitan University, Toronto, ON M5B 2K3, Canada.; ^2^ Department of Chemistry and Biology, Toronto Metropolitan University, Toronto, ON M5B 2K3, Canada.; ^3^ Synaesthetic Media Lab, The Creative School, Toronto Metropolitan University, Toronto, ON M5B 2K3, Canada.; ^4^ Department of Molecular and Cellular Biosciences, University of Southern California, Los Angeles, CA 90089, USA.

## Abstract

We describe the anatomy of replication forks by detecting proteins associated with DNA replication (Cdc45 and RPA), DNA damage (H2A.X), and DNA repair (Rad51) relative to tracts of synthesized, 5′-bromodeoxyuridine (BrdU)-labeled DNA on chromatin fiber images. These fibers track pixel intensity and positional data, which are analyzed using our program: R-ODD-BLOBS (One Dimensional Data Boolean Logic Operations Binning System in the programming language R). We studied the effect of threshold and signal smoothing for BrdU and protein tracts following hydroxyurea in wild type fission yeast (*Schizoasaccharomyces pombe*), compared to DNA replication checkpoint mutants *mrc1Δ* and *cds1Δ*. We show that R-ODD-BLOBS allows robust analysis of BrdU lengths and that Rad51, Cdc45, RPA, and H2A.X show distinctive, checkpoint-dependent locations around replicated tract ends. Rad51 was found at 22% more replicated areas in *mrc1∆* than in wild type, suggesting that homologous recombination repair may be more common at *mrc1Δ* forks. Helicase detachment in *cds1∆* post-HU was indicated by Cdc45 enrichment in unreplicated chromatin close to putative forks. Similarly, *cds1Δ* fibers show that Rad51, RPA, and H2A.X are distributed upstream of replicated areas more than in wild type. Excitingly, we find that H2A.X is distributed asymmetrically around replication forks, suggesting that the fork complex is a barrier for DNA damage signal extension into replicated areas. Together, R-ODD-BLOBS analysis shows a rigorous, iterative computational analysis tool to assess large chromatin spread datasets. R-ODD-BLOBS finds patterns of DNA replication length and protein components at replication forks that describe the anatomy of a fork and how structures change after replication checkpoint loss.

## Graphical Abstract

**Figure:**
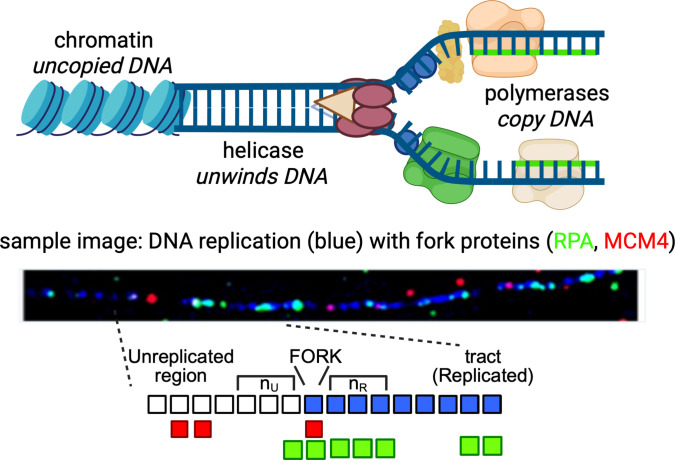

## Introduction

High-fidelity DNA replication complexes and DNA replication checkpoint mechanisms cooperate to maintain genome stability during DNA replication stress [1,2]. The functional units of DNA replication are replication fork complexes. Replication forks link helicase, polymerase, and checkpoint activities to ensure rapid and error-free synthesis [3–6]. After replication origins fire in S-phase, the Cdc45–MCM–GINS (CMG) helicase complex unwinds DNA template in front of each individual fork, generating single-strand DNA (ssDNA) [7,8] ahead of the leading (polymerase ε) and lagging (polymerases δ and 𝛼) strand polymerases [9–11]. The helicase-unwinding and polymerase-copying functions are structurally linked by the fork protection complex (FPC) [12,13]. Linking and stabilizing fork complex structures are important to prevent mutation and ensure complete genome synthesis, and are a barrier to diseases such as cancer [1,2].

The replication checkpoint controls cell cycle progression to prevent incomplete synthesis, responding to obstacles that slow or stall forks [14]. The replication arresting drug hydroxyurea (HU) depletes dNTP pools, causing fork slowing and stalling [15–17]. In the fission yeast, *Schizosaccharomyces pombe,* the replication checkpoint protein Cds1 (mammalian CHK1) is phosphorylated and activated by Rad3 kinase (mammalian ATR). Mediating this interaction is Mrc1 (mammalian CLSPN), a checkpoint protein that is also a structural component of the FPC that influences replication processivity [13,18–21]. Activated Cds1 during replication instability stabilizes forks when dNTPs decrease [22], prevents late-origin firing, and preserves the DNA replication program [17]. However, *cds1Δ* or *mrc1∆* mutant cells cannot activate a replication checkpoint and continuously synthesize DNA in HU stress. Forks collapse and generate DNA double-strand breaks (DSBs), cell elongation, and lethal G2 arrest [17,23]. An early symptom of fork collapse is replication protein A (RPA) recruitment to ssDNA at breaks. The histone variant H2A.X in mammals, H2A-S129 in yeast, is phosphorylated symmetrically around DSBs to promote DNA repair protein recruitment [24,25] (note: we refer to fission yeast phospho-H2A S129 phosphorylation as “H2A.X” for consistency in the rest of this manuscript). In the G2 phase, the Mre11–Rad50–Nbs1 complex and cofactor CtIP resect break ends and promote error-free repair through homologous recombination (HR) [26,27]. HR is dependent on Rad51 recruitment to the ssDNA, which displaces RPA [24,27] and allows homology search to promote repair from donor templates [27,28].

Replisome dynamics and anatomy during HU arrest have been extensively characterized using biochemical and molecular assays such as isolation of proteins on nascent DNA (iPOND) or chromatin immunoprecipitation (ChIP) and sequencing [29–31]. Since these methods average signals across whole-cell populations, called ensemble averaging, cell-to-cell or fork-to-fork heterogeneity may not be detected [32,33], and distinct fork subpopulations causing alternate phenotypes are lost [34]. While structural methods such as electron microscopy can image individual DNA replication forks and detect ssDNA, the forks are visualized outside of DNA–protein and genomic contexts and cannot track specific replisome components [11,35]. Understanding the diversity of replisome structure and effect relationships could be used to model fork stability mechanisms that predict instability and mutation.

Microscopy of linear chromatin fibers overcomes these limitations by preserving bound proteins for direct imaging of fork complexes across the genome. Performed in the context of a *cds1∆* or *mrc1∆* mutant strain following HU, chromatin fibers allow comparison of fork structures in conditions where DNA damage and death are known outcomes. Yet, the massive and multiparameter datasets generated by chromatin fiber images create an analysis and modeling challenge. To resolve this computational challenge, we developed the One Dimensional Data Boolean Logic Operations Binning System (ODD-BLOBS) to systematically correlate DNA synthesis (tract length) with protein location from chromatin fiber data [36]. Here, we show a robust, user-friendly, and open-source implementation of ODD-BLOBS in the programming language R (R-ODD-BLOBS). R-ODD-BLOBS automates processing of linear fiber data to detect replication tracts, smooth signal gaps, identify potential fork areas, and statistically map replisome proteins in viable (wild type [wt]) and at-risk (*cds1∆* and *mrc1∆)* forks [36]. As fork structure affects both synthesis amount and DSB generation [23], R-ODD-BLOBS can describe DNA–protein distributions and patterns at the level of individual forks.

We tested R-ODD-BLOBS by comparing nucleoside analogue-labeled new synthesis tracts on chromatin fibers from wt, *mrc1∆*, and *cds1Δ S. pombe* cultures after HU arrest. By correlating the spatial positioning of Cdc45, Rad51, RPA, and phospho-H2A.X relative to new synthesis, we detected distinct structural phenotypes across *cds1∆* and *mrc1∆* replication for subpopulations. We found that *mrc1∆* has the most Rad51 recruitment near fork areas, while *cds1∆* shows more Rad51 in unreplicated areas. We show Cdc45 detachment in over 40% of *cds1∆* forks. While wt and *cds1∆* RPA and H2A.X patterns are similar, we find that both DNA damage markers are asymmetrically distributed at replicated ends into unreplicated areas upstream of forks. This spatial analysis of multichannel fiber data by R-ODD-BLOBS shows variation of stalled replication forks between genotypes that may promote eventual checkpoint mutant death post-HU. We further show that the replication fork is a barrier to H2A.X spread into replicated areas. Thus, single-fork analysis of these asymmetric forks may provide clues to replisome failure and mutagenesis in checkpoint mutants.

## Materials and Methods

DNA synthesis was monitored using the nucleoside analogues 5′-bromodeoxyuridine (BrdU) and ethynyldeoxyuridine (EdU) as described previously (e.g., Refs. [22,23]). We used fission yeast strains (Table 1) that expressed the human equilibrative nucleoside transporter 1 (*hENT1+;* increases BrdU/EdU uptake into cells) and the Herpes Simplex Virus thymidine kinase (*hsv-tk+*); these transgenes convert BrdU/EdU into phosphorylated dNTPs for incorporation in DNA replication [22,23]. Cultures were grown in pombe minimal glutamate media (Sunrise Bioscience) with 225 mg/ml of adenine and uracil to mid-log phase (i.e., OD_600_ of 0.4 to 0.8, approximately 1 × 10^6^ cells/ml). HU (12 mM, BioShop, Canada) was added for 4 h at 32 °C with shaking to arrest cells in S-phase [23]. Cells were collected onto Whatman vacuum filters and washed twice with prewarmed medium before resuspension in an equal volume of medium. A nucleoside analogue was added to label DNA synthesis (either BrdU 50 μg/ml final concentration or EdU at 10 μM final concentration). Post-HU replication restart proceeded 30 min at 32 °C before cells were harvested for fiber preparation.

**Table 1.** Genotype and source of *S. pombe* strains

Genotype	Source
*h^+^ rad11myc::KanMX cdc45-YFP-ura4+ leu1-32::hENT1-leu1+(pJAH29) his7-366::hsv-tk-his7+(pJAH31) ura4-D18 ade6-M210*	FY 3805 ^a^
*h^+^ ∆mrc1::ura4+ cdc45-YFP-ura4+ leu1-32::hENT1-leu1+(pJAH29) his7-366::hsv-tk-his7+(pJAH31) rad11myc::KanMX ura4-D18 ade6-M210*	FY 3821
*h^+^ ∆cds1::ura4+ cdc45-YFP-ura4+ rad11myc::KanMX leu1-32::hENT1-leu1+(pJAH29) his7-366::hsv-tk-his7+(pJAH31) ura4-D18 ade6-M210 (rad11=ssb1)*	FY 3830
*h^−^ pola-FLAG2::ura4+ rad11myc::KanMX leu1-32::hENT1-leu1+(pJAH29) his7-366::hsv-tk-his7+(pJAH31) ura4-D18*	FY 3858
*h^−^ ∆mrc1::ura4+ pola-FLAG2::ura4+ rad11myc::KanMX leu1-32::hENT1-leu1+(pJAH29) his7-366::hsv-tk-his7+(pJAH31) ura4-D18*	FY 3859
*h^-^ ∆cds1::ura4+ pola-FLAG2::ura4+ rad11myc::KanMX leu1-32::hENT1-leu1+(pJAH29) his7-366::hsv-tk-his7+(pJAH31) ura4-D18*	FY 3866

^a^ Forsburg Yeast (FY) collection, SL Forsburg, University of Southern California.

Chromatin fibers were prepared as described in Ref. [36]. Briefly, 1 to 5 × 10^6^ cells were collected and arrested by incubating with 0.2% sodium azide on ice for 5 min. The cells were centrifuged and then washed with 1 culture volume of water. Pelleted cells were resuspended in zymolyase mixture (1 M sorbitol, 60 mM EDTA, 100 mM sodium citrate, 0.5 mg/ml zymolyase 20T [Novozyme], 1.0 mg/ml *Trichoderma harzianum* lysing enzymes [Sigma-Aldrich, Canada], and 100 mM 2-mercaptoethanol, pH 6.9) for 15 to 30 min. Spheroplasting was checked by microscopy. Spheroplasts were pelleted by centrifugation at 500 *g* for 5 min, then each spheroplast pellet was resuspended in 500 μl of phosphate-buffered saline (PBS).

Spheroplasts were adhered onto poly-lysine-coated coverslips (#1.5) in lines spaced 1 cm apart. Warmed lysing buffer (50 mM Tris-HCl, pH 7.4, 25 mM EDTA, 500 mM sodium chloride, and 0.1% Nonidet P-40 [Sigma], 0.5% [w/v] sodium dodecyl sulfate [BioShop], and 3 mM 2-mercaptoethanol; 70 °C) was pipetted onto each line and then incubated for 15 min to burst spheroplasts. Coverslips were tilted at a 10° to 25° angle to run lysate down the slide, extending and stretching the fibers. Samples were air-dried vertically at room temperature and then treated with fixative (4% paraformaldehyde in PBS) for 10 min in the dark. Samples were rinsed in PBS and then baked for 5 to 10 min at 60 °C on a warming plate. Samples were stored flat at −20 °C until staining.

### Fiber labeling methods to detect synthesis and proteins

To detect BrdU, fibers were first wet in PBS and then denatured in 2 M HCl for 15 min. Samples were neutralized in 0.1 M sodium tetraborate (Na_2_B_4_O_7_; BioShop Canada) solution (pH 8.5) for 10 min, and then washed in PBS. Slides were blocked in the Blocking buffer (10% fetal calf serum, 10% bovine serum albumin, and 0.05% Tween 20 detergent in PBS and filter sterilized) in a dark, humid chamber. BrdU was detected with rat BU1/75 (ICR1, Abcam), diluted to 1:100 in the blocking buffer.

To detect EdU, fibers were first wet in PBS. Coverslips were then incubated for 5 min in PBB (1× PBS + 0.5% BSA, filter sterilized) at room temperature before click detection. Samples were incubated on coverslips with 50 μl of freshly prepared ClickIT-EdU reagent, made according to directions (Thermo Fisher Scientific, Molecular Probes, USA). We have used kits with different fluors, Alexa Fluor 488, Alexa Fluor 647, Alexa Fluor 555, and picolyl Alexa Fluor 488, with equal results. ClickIT mixture was applied to fibers on coverslips and incubated under a parafilm cover in a humidified chamber for 30 min at 37 °C. Samples were washed 3 times with PBB, and then once with PBS. Samples were mounted onto glass slides using approximately 5 μl of SlowFade mountant with 4′,6-diamidino-2-phenylindole dihydrochloride (DAPI; Molecular Probes).

To detect proteins, BrdU or EdU detection was performed first. Following primary antibody application or Click reaction, antibodies against epitope tags were added in the Blocking buffer. Cdc45-YFP was detected with anti-GFP antibody (Abcam, 1:500). RPA-myc was detected with anti-myc antibody (9E10 clone, Covance, 1:500). FLAG-polymerase alpha (Pol𝛼) was detected with anti-FLAG M2 (Sigma, 1:500). Rad51 was detected using a rabbit polyclonal anti-Rad51 serum generated by the Forsburg lab (1:500). Primary antibodies were applied to coverslips, covered with a parafilm cover, and incubated overnight at 4 °C in the dark. Samples were washed in PBS 3 times, and then secondary antibodies were applied and incubated at 30 °C for 2 h. Secondary antibodies against each primary antibody were diluted in blocking buffer at 1:500, including chicken anti-rat Alexa Fluor 488 (Invitrogen), goat anti-rabbit Alexa Fluor 546 (Invitrogen), and donkey anti-mouse Alexa Fluor 647 (Invitrogen). Samples were washed in PBS and mounted onto glass slides with SlowFade Gold antifade mount containing 1 μg/ml DAPI (Invitrogen).

### Fluorescence *in situ* hybridization methods to calculate fiber stretching

Ahead of time, a GFP-labeled cosmid probe was prepared using a nick translation kit (Roche, USA). Briefly, cosmid c1095 was linearized by digestion, and the nick translation kit was used to synthesize probes. The c1095 probe was confirmed by checking size and integrity on a 1% agarose gel. The c1095 probe was precipitated using sodium acetate and ethanol, washed in ethanol, and then resuspended in 1× TE (10 mM Tris-HCl pH 8, 1 mM EDTA). Before hybridization, the probe was denatured at 70 °C for 15 min and then kept in a heated block until ready to use on fiber samples.

Fiber samples on coverslips were processed to detect BrdU and proteins using primary and secondary antibodies as in the “Fiber labeling methods to detect synthesis and proteins” section. Antibodies were crosslinked to fibers by incubating samples in 4% formaldehyde in 1× PBS + 0.05% Tween-20 for 10 min, then washing in 1× PBS + 0.05% Tween-20. Coverslips were dipped in SSCT buffer (2× SSC + 0.1% Tween-20, pH 7.0), and then pretreated for denaturation for 20 min at 37 °C in SSCT in a humidified chamber. The DNA on fiber coverslips was denatured in 70% formamide/SSCT at 75 °C for 15 min, and then a denatured probe was applied. The samples with probes were sealed onto a prewarmed glass slide with rubber cement, and the samples were incubated in the humidified chamber at 37 °C for 24 to 48 h. Hybridized samples were washed 4 times in 50% formamide/SSCT at 45 °C to remove nonspecific probes. Excess probes were removed from coverslips with 4 washes in SSCT at 37 °C for 2 min each, and then 4 washes in room temperature SSCT for 5 min each. Samples were mounted onto slides counterstained with SloFade Gold + DAPI mount (ThermoFisher, CA).

### Imaging spread fibers

Samples were imaged using a DeltaVision microscope with softWoRx v4.1 (GE, Issaquah, WA) using a 60× (numerical aperture [NA] 1.4 PlanApo) lens, solid-state illuminator and 12-bit charge-coupled devicecamera. Images were acquired in 5 0.2-μm z-sections, then deconvolved and maximum intensity projected (softWoRx, default settings). Image stacks were deconvolved and projected. Fibers were manually traced by drawing 1-pixel (px)-wide line segments with the “Arbitrary Line Profile” tool (softWoRx, Applied Precision Instruments), and fluorescent intensity values from each channel were acquired. Data were processed into tab-delimited files for analysis with R-ODD-BLOBS. A minimum of 3 fields per strain were analyzed, and the data were compared among 3 or more experimental replicates.

### R-ODD-BLOBS data processing and analysis

We ran the intensity data using R-ODD-BLOBS (download link: https://github.com/SabatinosLab/R-ODD-BLOBS; Table 2) to visualize initial scatter plot data (as shown in Fig. S1) and estimate preliminary baseline threshold values. The preliminary baseline threshold was determined by selecting the lowest value from a cluster of intensity data (Fig. S1), thereby removing low-value outliers. We reran R-ODD-BLOBS by fixing 2 channels with baseline threshold values, and iterating the dependent variable from thresholds of 100 to 1,000, increasing by increments of 100 (all fluorescent intensity measurements are in arbitrary units). We found that optimal thresholding values across multiple samples/experiments of our first dataset were DNA at 100, and BrdU, Rad51, and Cdc45 at 200. For our second data, our thresholding values were DNA, BrdU, and RPA at 300, and H2A.X at 400.

**Table 2.** R-ODD-BLOBS Github link

Program	Link
R-ODD-BLOBS	https://github.com/SabatinosLab/R-ODD-BLOBS

After thresholding, we used a smoothing effects analysis to test smoothing values from 0 to 6, with optimal threshold values found above. The best smoothing value for our first data was 3 for DNA and BrdU, and 4 for Rad51 and Cdc45. Our second dataset had similar values, 3 for DNA and BrdU, and 4 for RPA and H2A.X. After the threshold and smoothing were determined, R-ODD-BLOBS was run using the optimized values to determine protein locations in potential fork regions (i.e., unreplicated, fork, and replicated) and to calculate the length of BrdU or Cdc45/Rad51/H2A.X/RPA tracts.

## Results and Discussion

**Fig. 1.**
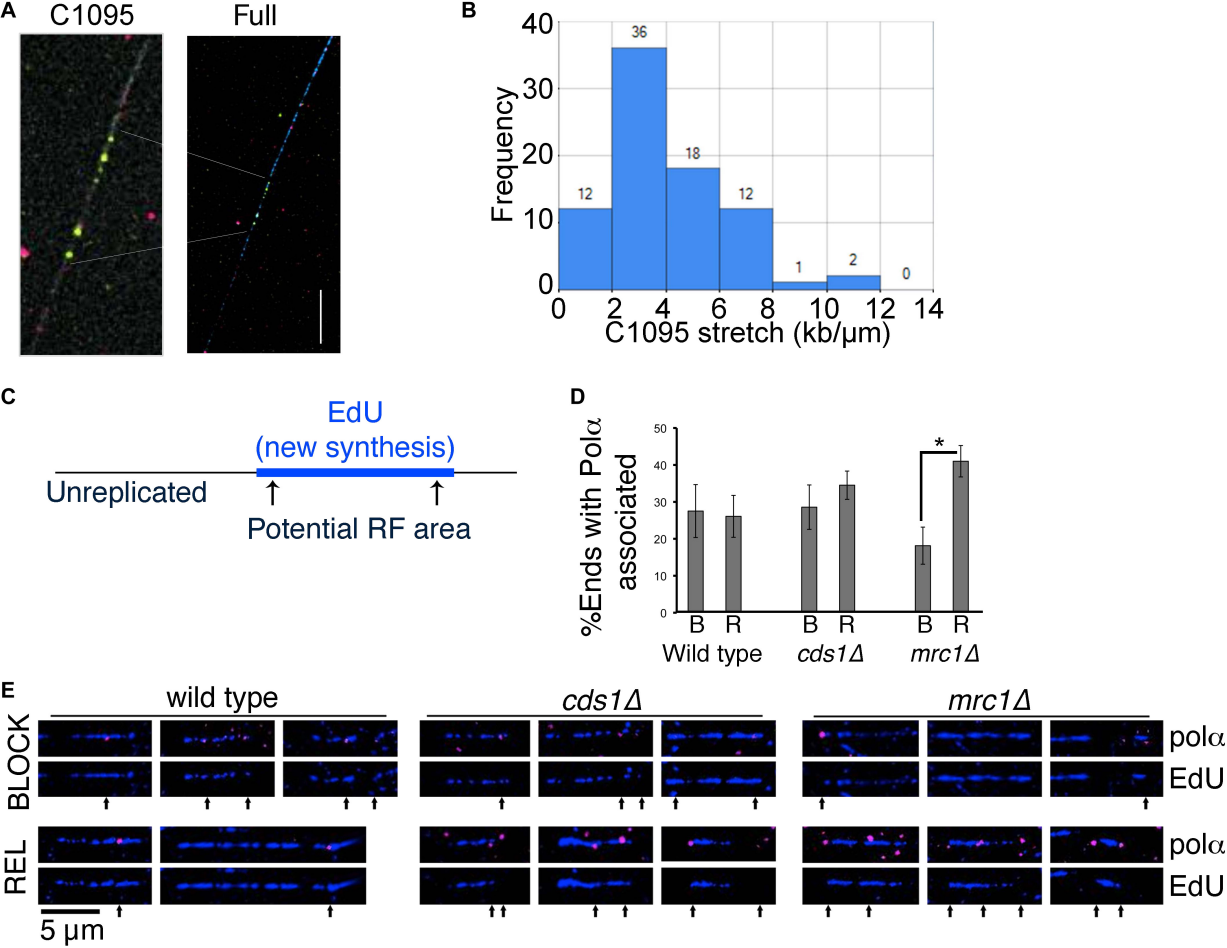
Pol𝛼 association with replicated tracts is similar during HU release in wild type and *cds1∆, mrc1∆* checkpoint mutants. (A) Microscopy image of a chromatin spread fiber with a fluorescence *in situ* hybridization (FISH) cosmid probe to detect the c1095 area of the genome. Left: Zoomed-in view shows c1095 FISH probe (green) with DAPI chromatin fiber signal (gray) and red MCM2 helicase. Right: The full fiber shown with BrdU label (blue), MCM2 (red), and c1095 FISH probe (green); scale bar, 10 μm. (B) Histogram of c1095 distances, converted to kb/μm. Data from *n* = 81 c1095 tracts across 5+ images in each of 3 experiments were measured in pixels from end to end. c1095 tracts were converted to kb/μm using the known c1095 distance of 28.6 kb. Fiber stretch centers between 2 and 4 kb/μm, and the mean value for fiber stretch was calculated at 3.1 kb/μm. (C) Scheme for analyzing replicated tracts on spread DNA fibers. Each replicated tract has an internal (replicated) zone and external unreplicated areas. We defined the ends of each synthesized tract as potential replication fork areas. (D and E) Newly synthesized tracts were detected by adding EdU nucleoside analogue during HU block (4 h, 12 mM HU at 32 °C) and release (30 min, 32 °C). EdU was detected with Click chemistry, and DNA polymerase 𝛼 (Pol𝛼-FLAG) was detected using anti-FLAG-M2 and fluorescent secondary antibodies. (D) EdU tracts were assessed by eye to detect Pol𝛼-FLAG near tract ends. Tracts shown are sample EdU tracts around EdU incorporated tracts that may have replication forks. EdU ends with Polα are presented as the percent of total EdU tract ends examined during the HU block (labeled B) or following release from the drug (labeled R). Proportion shown with 95% confidence interval from 2+ replicates. (E) Shown are sample EdU tracts around microscopy images of Pol𝛼 (pink) at EdU tract ends (tag in blue) during 4 h HU (BLOCK) and at 30 min after release from HU (REL) as quantified in (D). EdU tracts are shown below the merged image. Pol𝛼 in potential replication fork areas is indicated by arrows under the tracts.

R-ODD-BLOBS analysis was designed to develop chromatin fiber methods that can be used to detect replicated areas and associated proteins [37–39]. Chromatin fibers are influenced by stretching during preparation, which might influence length of DNA synthesis tracts, so we used a fluorescence *in situ* hybridization (FISH) probe to measure the average length of a constant. A 28.6-kb cosmid C22G7 (c1095, 0.726 to 0.755 Mb) was labeled and hybridized to fibers; this detects chromosome 1 within 10 kb of an early and strong origin, ORI 12 [10,40,41]. We found a mean of 9.2 μm per probe signal, indicating approximately 3.1 kb/μm of DNA extension (standard deviation, 2.12 kb/μm) (Fig. 1A and B). With a pixel size of 0.1092 μm/px in our microscopy system, this converts to approximately 331 bp/px in our images. With this image (px)-to-DNA (bp) conversion value in mind, we continued to analyze replication tracts and proteins using pixels as the smallest, but easily convertible, unit of measurement.

### Pol𝜶 at replicated tract ends in checkpoint mutants

Although *cds1*∆ and *mrc1*∆ mutants continue DNA synthesis during and after HU arrest [23], the same cells accumulate DNA damage/DSB signals and are unable to form colonies after 4 h in HU. This paradox—that cells undergoing replication collapse still show functional fork activity—might be explained if *cds1∆* and *mrc1∆* forks retained polymerases because structural decay and collapse occur later. We used thymidine analogue EdU to label new DNA post-HU and defined the ends of EdU tracts as potential replication fork areas at 30 min after HU release (Fig. 1C). Because chromatin fibers retain proteins during preparation [39], we used a FLAG antibody to detect FLAG-pol𝛼 on fibers (Fig. 1D and E). Comparing FLAG-pol𝛼 near EdU tracts at 4 h in HU (BLOCK), or 30 min after release (REL), we found that approximately 30% of EdU tracts had Pol𝛼 near an end in both wt and *cds1∆* (Fig. 1D and E; FLAG-pol𝛼 are pink foci on blue EdU tracts). The *mrc1*∆ tracts had less nearby Pol𝛼 in BLOCK but regain Pol𝛼 during release. Because Pol𝛼 acts discontinuously on the lagging strand, we conclude that wt and *cds1∆* share similar Pol𝛼 retention mechanisms during and after HU. However, *mrc1∆* forks are more likely to lose Pol𝛼 during HU; we predict that this results from FPC instability without Mrc1. Together, Pol𝛼 decoration at tract ends shows that forks might be compared using chromatin fibers and that Mrc1/FPC promote lagging strand stability during replication stress.

### Thresholding analysis in R-ODD-BLOBS provides rigorous comparison of signal inclusion/exclusion

Our confirmation of replication fork protein localized at the ends of replicated tracts formed a basis for the next steps of computational analysis using R-ODD-BLOBS from chromatin fibers (Fig. S1A). The previous ODD-BLOBS software to analyze chromatin fibers used a Visual Basic for Applications script [36]. While ODD-BLOBS ran on both Excel and LibreOffice, the interface was difficult to use, and processing was time-intensive. We adapted ODD-BLOBS into an R script to make R-ODD-BLOBS (see Table 2). R-ODD-BLOBS first makes a scatter plot of initial intensity values (Fig. S1B), which we used to estimate a preliminary baseline threshold for each fluor/channel. This step also compared fluorescent signal intensity and staining differences between images to find failed labeling or high-background images.

**Fig. 2.**
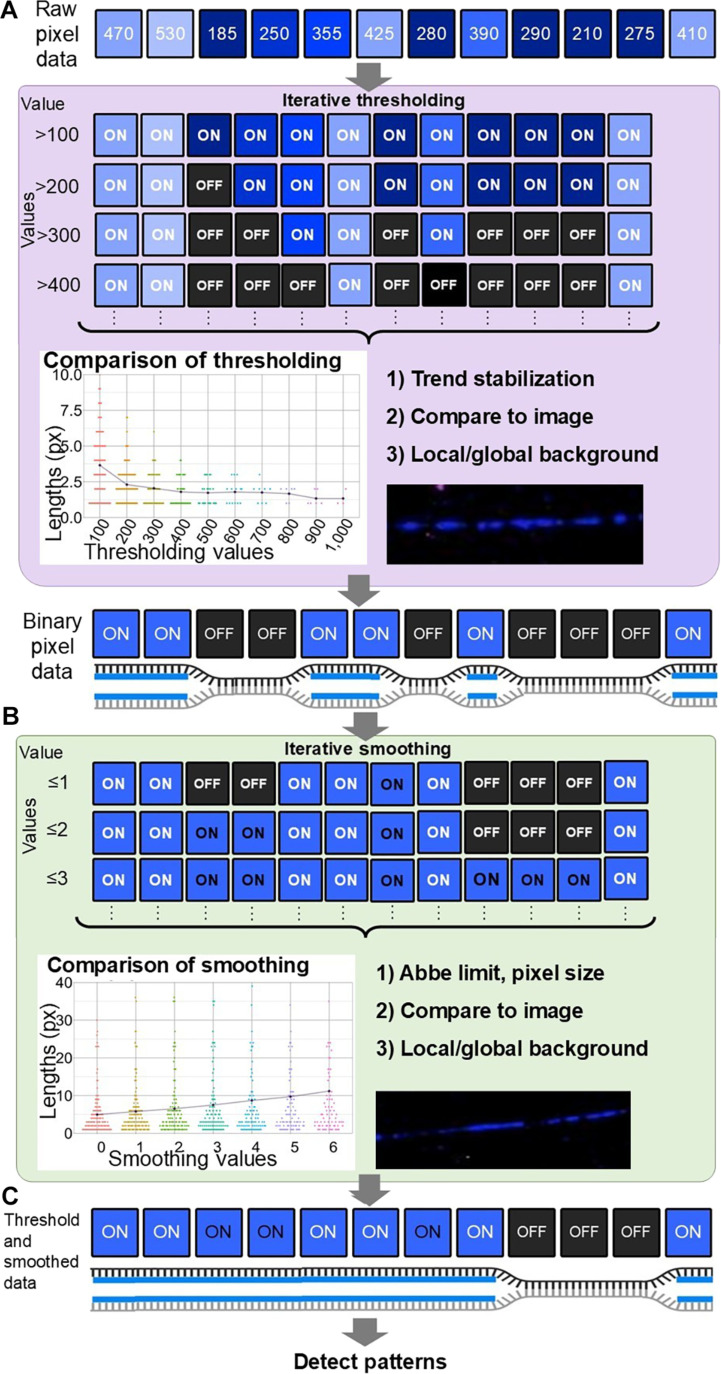
Using and optimizing the thresholding and smoothing pipeline of R-ODD-BLOBS. R-ODD-BLOBS thresholding and smoothing functions allow users to adjust how the program processes their data and test parameter settings on data output. Smooth-it allows users to “smooth” small gaps in pixel-intensity data. (A) Thresholding defines the minimum pixel intensity included in the data. A sample of initial raw fluorescent intensity data of a single channel is shown for each pixel on a line. Iterative thresholding analysis (purple background) allows users to graphically compare the effects of thresholding on the data. The optimal threshold can be chosen by considering parameters including the following: (1) Trend stabilization: in the figure example, the inflection point at 300 defines pixels above 300 as “ON” and those below the threshold as “OFF”. (2) Visual comparison to the original image can be used to assess threshold choice relative to the actual image. (3) The local or global background signal can be sampled and used to define the minimum value of the true signal above a background (i.e., as in Ref. [23]). Thresholding generates binarized data of what is considered a true signal in the data (pixel-ON) and what is excluded in analysis (pixel-OFF). Below, a fork “cartoon” shows the impact of choosing the 300 threshold on binarized data, which can be followed by smoothing or detecting fork patterns. (B) Iterative smoothing analysis (green background) tests the impact of joining subresolution gaps in signal. A series of OFF pixels between 2 ON pixels, which is smaller than the smoothing value, would be converted to ON in the resulting dataset. Blue box pixels with white “ON” text are the original above-threshold ON pixels, while blue pixels with the black “ON” text were “smoothed” and joined the tracts. This simple iterative analysis for all fluorescent channels allows users to graphically compare the effects of smoothing on their BrdU and protein data; other more intensive approaches could also be used before importing data into R-ODD-BLOBS for replication fork window analysis (i.e., Refs. [42–45]). The choice of smoothing value can be guided by the effect on tract length and considerations including the following: (1) The Abbé limit, as the limit of light resolution captured in a microscope system; thus, any gaps smaller than those values are not confidently separated. (2) Visual assessment, to compare gaps in signal with the microscopy image. (3) Background assessment. (C) After threshold and smoothing, the data can be used to define replicated tracts and tips, and correlate with proteins that are processed for threshold/smoothing using their own parameters. Signals in all image channels are registered along the line of pixels and spatially organized by EdU/BrdU replicated areas and tips. Because the pixel size of a microscope is a defined distance, the inclusion/exclusion of proteins can be examined around potential fork zones at replicated tract tips.

**Fig. 3.**
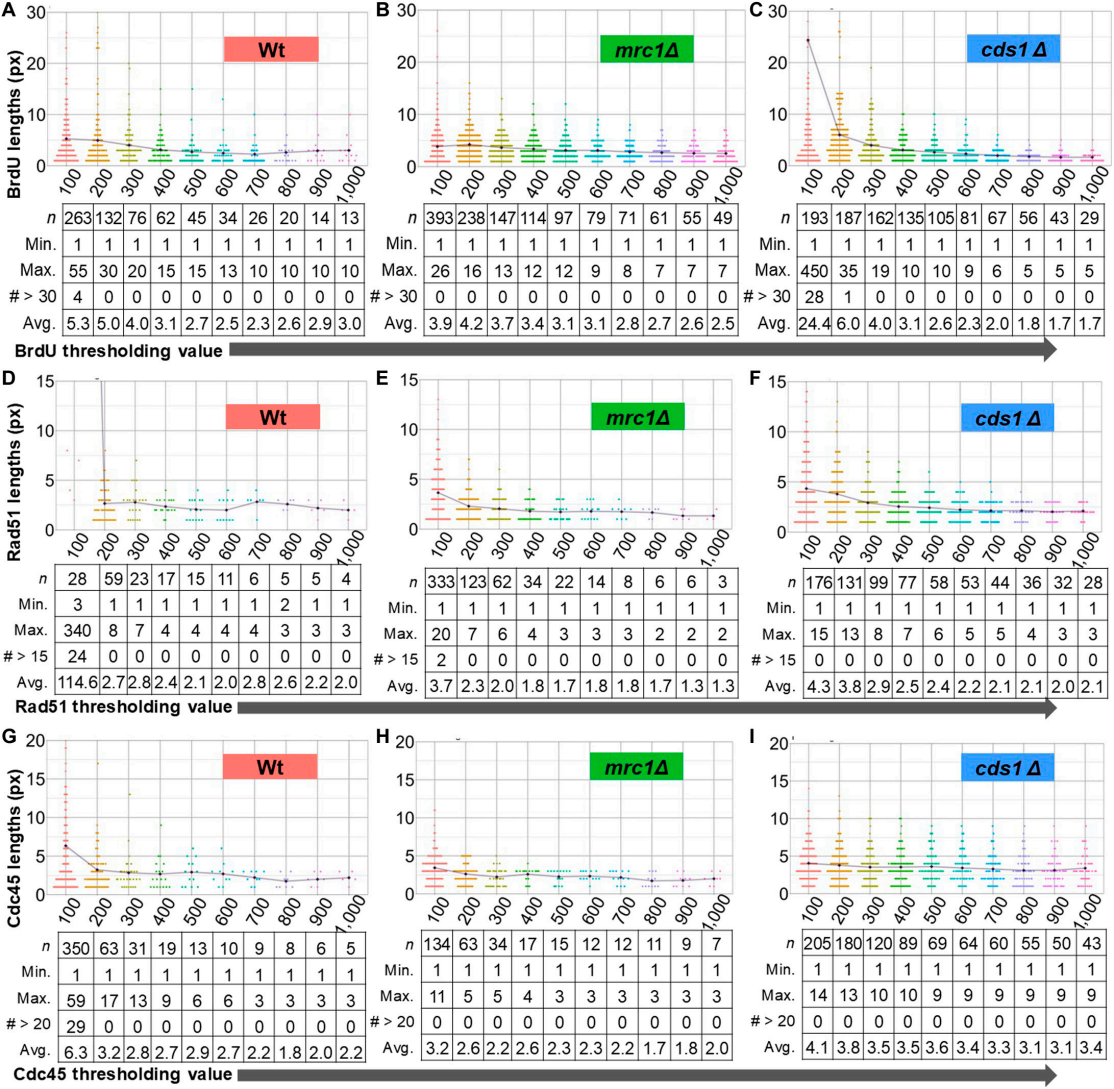
Increased threshold values decrease BrdU, Rad51, and Cdc45 lengths. The intensities of chromatin fiber data (2+ experiments, 5+ images per experiment) were processed using R-ODD-BLOBS for thresholds from 100 to 1,000 to remove background signal. DNA synthesis (BrdU) and replication-associated protein (Rad51 and Cdc45) channels were iterated separately, but conserving the location along the pixel register line. The value of 100 arbitrary units was chosen as a baseline threshold based on visual inspection of intensities in a scatter plot (i.e., Fig. S1), calculated as 100 for DNA, 150 for BrdU, 120 for Rad51, and 130 for Cdc45. While upper tract lengths are fixed for each label (30 for BrdU, 15 for Rad51, and 20 for Cdc45), outliers are shown as the maximum values in the chart below each threshold bin. Individual thresholding analysis for each replicate is shown in Figs. S2 to S4. Average tract length values are shown as a point in each threshold column and are connected by a linear fit. (A to C) Bee swarm plots of BrdU tract lengths in wt, *cds1Δ*, and *mrc1Δ* at thresholds from 100 to 1,000. (D to F) Bee swarm plots of Rad51 signal lengths in Rad51 in wt, *cds1Δ*, and *mrc1Δ* at thresholds from 100 to 1,000. (G to I) Bee swarm plots of Cdc45 signal lengths in wt, *cds1Δ*, and *mrc1Δ* at thresholds from 100 to 1,000.

As signal inclusion/exclusion becomes increasingly important for detecting fork proteins to assess biology, we developed a more rigorous analysis to identify optimal thresholds for each signal (Fig. S1C and D). We iterated analysis using threshold values from 100 to 1,000 for BrdU, Cdc45, and Rad51 and found tract lengths and number of tracts (Fig. 2A). As predicted, we found that higher thresholds exclude background and outlying data, resulting in shorter tracts. As BrdU threshold increased, fewer pixels were above threshold values, and the length of tracts decreased because of gaps in signal (Fig. 3A to C). We found that there is a point where the effect of threshold increase is minimal. For example, a threshold of 200 in the sample BrdU data of Fig. 2A shows that length variation is minimal above the 200 threshold; this is close to the 150 threshold that was assessed from the initial scatter plot (Fig. S1B).

Wt BrdU tract lengths plateaued above a threshold of 300 (Fig. 3A). Average BrdU lengths of *mrc1∆* fibers were shortest and least affected by threshold increase (Fig. 3B). BrdU tracts were longest in *cds1∆* samples and were most impacted by threshold increases (Fig. 3C). The saturation point of threshold effect on average length was consistent between replicate sample populations (Fig. S2). Because thresholding could be impacted by image background intensity or outlying nonspecific signals (Fig. S10), R-ODD-BLOBS linear analysis excludes pixel intensities that are less than the user-input values (demonstrated in Fig. 2A). While this may lead to small gaps, smoothing (Fig. 2B) can connect subresolution gaps before patterns are detected (Fig. 2C and Fig. S1C).

Thresholding effects on protein signals were similar across genotypes: increased threshold values tended to decrease tract length, although Rad51 and Cdc45 tracts reach a minimum signal that is less impacted by threshold increase than BrdU (Fig. 3D to I and Fig. S3). Average Rad51 tracts of any genotype did not change above a 300 fluorescent intensity-threshold setting. Cdc45 tract lengths also showed little change between thresholds (Fig. 3G to I and Fig. S4). A simple conclusion is that Cdc45 and Rad51 occupy less space on fibers, compared to the long tracts of BrdU during recovery from HU; importantly, Cdc45 and Rad51 signals can be correlated with visual inspection of images (i.e., Fig. S1D).

### Smoothing extends tracts and minimizes signal gaps

Small pixel gaps that result from better threshold discrimination in an otherwise continuous tract raise a question: is the loss of signal a true gap in replication/protein, or do gaps reflect signals that are slightly below the strict level of a threshold and not “real” from the perspective of resolution? To test the second possibility, we implemented a simple smoothing algorithm that connected tracts separated by subresolution gaps. The Abbé Limit guides smoothing for signal gaps below the limit of light resolution. Using a green light excitation (488 nm), NA, and pixel size for the system (1.4 NA, 0.1092 μm/px), the Abbé limit states that gaps in BrdU signal less than 3 px long cannot be discriminated against as truly absent. The “Smooth-It” [36] function connects subthreshold “OFF” pixels that are between 2 groups of “ON” pixels (Fig. 2B); thresholding and Smooth-It function together to assess data quality and the effect sealing small, subthreshold gaps (Fig. 2C) providing defined parameters in hypothesis testing.

**Fig. 4.**
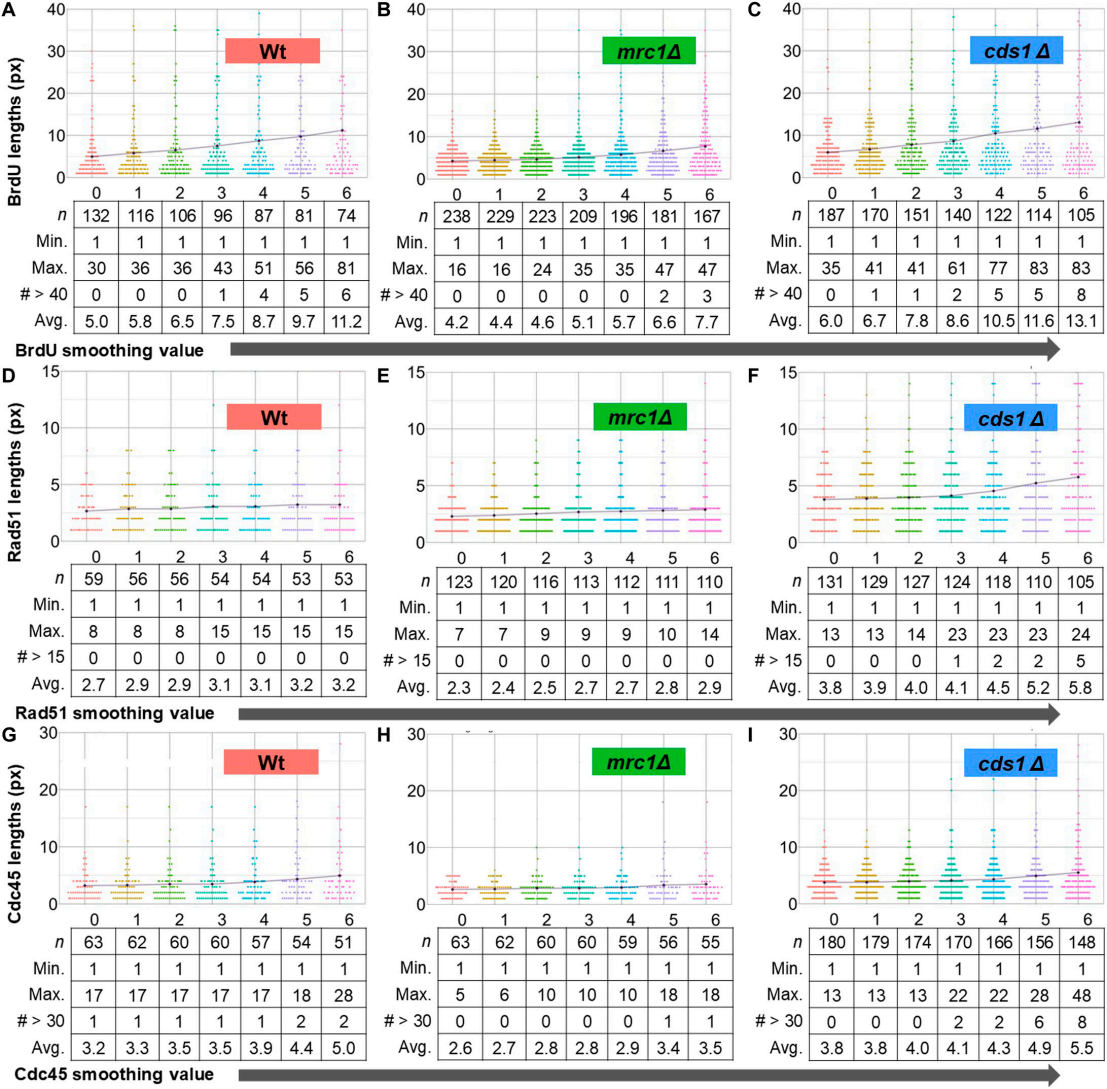
Regardless of genotype, larger smoothing values generate longer tract lengths for BrdU, Rad51, and Cdc45. The intensity of the line-traced chromatin fiber data was processed using R-ODD-BLOBS using previously determined threshold values: 100 for DNA; 200 for BrdU, Rad51, and Cdc45. Smooth-it links tracts together if the number of pixels is equal to or less than the “smoothing value” used. Average tract length values are shown as a point in each threshold column and are connected by a linear fit. (A to C) Bee swarm plots of the tract lengths in BrdU in wt, *cds1Δ*, and *mrc1Δ* at smoothing values 0 to 6 px. (D to F) Bee swarm plots of the tract lengths in Rad51 in wt, *cds1Δ*, and *mrc1Δ* at smoothing values 0 to 6 px. (G to I) Bee swarm plots of the tract lengths in Cdc45 in wt, *cds1Δ*, and mrc1Δ at smoothing values 0 to 6 px. Individual smoothing analysis for each replicate is shown in Figs. S5 to S7.

While more robust gap joining methods are implemented in other fiber-labeling procedures [42–45], we used an iterative approach to test the impact of gap joining on both BrdU and Cdc45/Rad51 signals for the purpose of streamlined import to fork window assessment. We smoothed gaps in signal of 1 to 6 px and compared tract lengths (Fig. 4). As predicted, smoothing increased tract lengths of BrdU and protein signals. Although 6 px smoothing doubled average BrdU tract lengths of wt and *cds1∆* samples (Fig. 4A and C), BrdU in *mrc1∆* was less impacted by smoothing (Fig. 4B). The Cdc45 tract lengths were unchanged by smoothing 1 to 6 px gaps in all genotypes (Fig. 4G to I), suggesting that Cdc45 is a single “point” protein that does not spread into larger complexes; this is consistent with literature showing that Cdc45 and the CMG complex are discrete and do not multimerize [46,47].

Rad51 forms polymeric filaments that coat ssDNA and promote repair during HR and fork regression restart [48–51]. HR is a preferred error-free method of repair during S-phase [52], and Rad51 is detected at replication forks [48,50,53,54]. Because *cds1∆* and *mrc1∆* show more phospho-H2A DNA damage signals after 4 h in HU, we predicted that Smooth-It would generate longer Rad51 tracts in replication stability mutants*.* Smoothing 1 to 6 px as above, we found that Rad51 tracts become longer in *cds1∆* but not in wt or *mrc1∆* (Fig. 4D to F). Longer Rad51 tracts in *cds1∆* are consistent with reports that Rad51 is regulated by Cds1 during HU arrest and restart [49]. While average *cds1∆* lengths of Rad51 vary from 3.8 px (no smoothing) to 5.8 px (6 px smoothing), the increase in Rad51 tracts greater than 15 px suggests Rad51 filament-associated fork structures. We conclude that Smooth-It seals signal gaps that create inappropriate subresolution protein detection sites and allows better Rad51 protein analysis.

### BrdU, Rad51, and Cdc45 tracts are all longest in cds1∆ post-HU

**Fig. 5.**
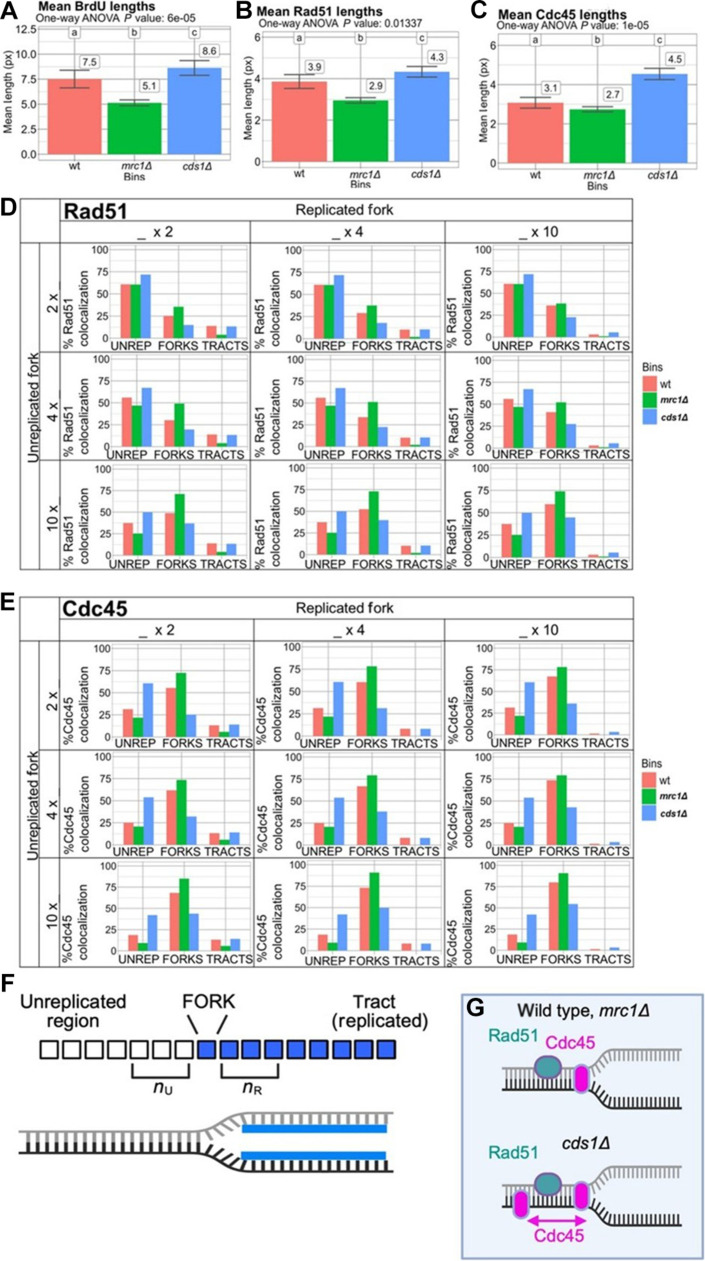
Fork window analysis of BrdU, Rad51, and Cdc45 tracts shows differences between wild type, *mrc1∆*, and *cds1∆* forks during HU restart. Thresholding and smoothing values determined from Figs. 3 and 4 were used to determine tract lengths of BrdU and the proteins Rad51 and Cdc45 using R-ODD-BLOBS. Cells were treated with 12 mM HU for 3.5 h, then released into fresh medium containing BrdU to detect BrdU incorporation after 30 min. All values are shown in pixels (px). Data from 3 experimental replicates were used for each of the wt, *cds1Δ*, and *mrc1Δ* datasets. Variation in tract lengths was compared using a 1-way ANOVA with Tukey’s HSD post-hoc correction in (A) to (C). (A) Average BrdU-tracts post HU are longest in wt and *cds1∆,* but shorter in *mrc1∆.* This is consistent with previous research [23]. There is significant variation between genotypes (*P* < 6 × 10^−5^). (B) Rad51 recombination protein tracts are longest in *cds1∆* tracts after HU and shortest in *mrc1∆* (*P* < 0.01). (C) Cdc45 lengths are similar in wt and *mrc1∆*, and longer in *cds1∆* (*P* < 1 × 10^−5^). (D) Bar graphs compare iterated pixel numbers around tips relative to Rad51-region coincident (related to Fig. S8). Proteins that are colocalized with replicated pixels are labeled “TRACTS”. Proteins that are colocalized at a tract tip (or within the *n*_U_/*n*_R_ window) are labeled “FORK”. Proteins that were detected on a chromatin fiber but are not colocalized with BrdU synthesis are unreplicated “UNREP”. With a 2 × 2 window (2 pixels included as FORK on either side of the tract tip), Rad51 is largely unreplicated (“UNREP”), regardless of genotype. As the number of unreplicated pixels included with the tract tip increases from 2 to 10 (unreplicated fork iterations, down), more Rad51 is colocalized with FORKS. As the number of replicated pixels included with the tract tip increases from 2 to 10 (replicated fork iterations, left to right), Rad51 distribution at FORKS increases slightly, suggesting that replicated Rad51 distribution is close to the tips. A full analysis of 2 px steps is shown in Fig. S8. Rad51 levels at forks are highest in *mrc1∆*; wt Rad51 is near FORKS. In contrast, *cds1∆* forks retain the most Rad51 in unreplicated areas that are away from forks, suggesting that DNA damage spread out away from collapsing forks. The 10 × 10 extreme situation (bottom right) is dominated by the effect of unreplicated pixels made fork proximal. (E) Analysis of Cdc45 distribution around replicated tract tips as performed for Rad51 in (D). A full iterative analysis in increments of 2 px is found in Fig. S8. Cdc45 is mostly unreplicated in *cds1∆* and is mostly tip-located at the fork in wt and *mrc1∆*. This pattern was found across all iterations; however, the proportion differs slightly when replicated forks are larger than unreplicated forks. (F) The effect of increasing/decreasing associated pixel numbers around a tract tip is shown. Each box represents replicated (blue) or unreplicated (clear) pixels after thresholding and smoothing. The last replicated pixel “tip” is where a fork may be found. By iterating the number of pixels included as part of the fork in either the unreplicated (*n*_U_) or replicated (*n*_R_) areas, R-ODD-BLOBS can test protein colocalization patterns. In wt cells, we predict that most Cdc45 will be close to the tip of the replicated tract; Cdc45 may move into unreplicated areas in *cds1∆* and *mrc1∆,* preceding fork collapse. wt cells are expected to have Rad51 in small tracts that may be in unreplicated areas following DNA damage; in *cds1∆,* Rad51 appears to accumulate more in unreplicated areas away from the fork. (G) Summary of Rad51 and Cdc45 placement around replicated fork windows in wt, *mrc1∆,* and *cds1∆* cells after HU treatment and 30 min recovery. We found that Rad51 in all strains is mostly unreplicated in all samples if there is no tip window, but unreplicated Rad51 becomes fork coincident with increased pixels around tract tips.

We next used R-ODD-BLOBS to calculate average tracts during restart after 4hHU. This ensemble averaging estimated the strongest trend within a population and is analogous to iPOND or ChIP sequencing results. Consistent with earlier findings that BrdU tracts differ between genotypes [23], we found that average *cds1∆* HU-restart tracts of BrdU were longest (8.6 px, or ~2.9 kb), wt at 7.5 px (~2.5 kb), and *mrc1∆* were shortest (5.1 px, ~1.7 kb) (Fig. 5A). Because data were not filtered for a minimum tract length, these average tracts are influenced by shorter signals; future analysis will assess whether a minimum length is optimal for fork complex detection.

Because there is more damage in replication checkpoint mutants post-HU [23], we hypothesized that there would be more Rad51 in *cds1∆* cells. We found longer Rad51 tracts on *cds1∆* fibers compared to *mrc1∆* (Fig. 5B). We also found that Cdc45 signals were longer in *cds1∆* (Fig. 5C) than either wt or *mrc1∆.* A general pattern was that average BrdU, Rad51, and Cdc45 tracts were longest in *cds1Δ,* followed by wt and then *mrc1∆.* Because Cdc45 is likely a point, and Rad51 lengths in pixels were similar to Cdc45, the aggregate data indicate that most Rad51 tracts are not particularly long. Further, the *mrc1∆* Rad51 and Cdc45 average tracts were less than the Abbé limit (3 px).

### Rad51 and Cdc45 are altered at cds1∆ and mrc1∆ tracts

We next examined BrdU tract ends to detect Rad51 and Cdc45 in potential fork areas. R-ODD-BLOBS classifies fork-coincident proteins by first defining the 2 ends of BrdU replicated tracts and labeling each tip as a potential “fork”. The unreplicated section of the fiber is pixels that are not labeled with BrdU, only with DAPI. The replicated section of a fiber is BrdU labeled and located between 2 “fork” tips. These 3 elements (illustrated in Fig. 5F) make up the fork analysis window. First, we used optimized thresholds to identify tracts. Since smoothing affects both the length of protein/BrdU and its location, we tested how smoothing affects each of BrdU, Cdc45, or Rad51 independently or for protein-BrdU colocalization effects. Previously determined smoothing baseline values at each channel’s Abbé limit (BrdU = 3, Rad51 = 4, Cdc45 = 4) were compared to no smoothing (=0 for each channel; Fig. S8A). We found that iterated smoothing of BrdU with Rad51 or Cdc45 had no effect on protein detection at unreplicated areas, tract tips (fork zones), or replicated regions within each sample set.

Yet, BrdU smoothing decreased Rad51 and Cdc45 fork detection in *cds1∆* and increased Rad51 and Cdc45 detection in replicated areas (Fig. S8A). We proceeded with optimized threshold and baseline smoothing values, and found that most Rad51 was in unreplicated areas (Fig. S8B). Intriguingly, 20% to 28% of Rad51 in wt and *mrc1∆* was at replicated “fork” tips, and only 3% to 15% of Rad51 was found in BrdU-replicated areas. We predicted that replicated areas away from the tract tip do not have active fork complexes and that the replisome is more likely to be distributed from a tip into nearby, unreplicated chromatin. Using the same methods, we found that Cdc45 is largely absent in replicated areas, particularly in *mrc1∆* (Fig. S8C). Instead, most Cdc45 is at “fork” tract tips in *mrc1∆* and wt. Most Cdc45 is in *cds1∆* unreplicated regions. Coupled with our earlier visual detection of Pol𝛼, our optimized R-ODD-BLOBS analysis indicates that *mrc1∆* forks regain Pol𝛼 and have Cdc45 association like wt with limited Rad51. The *cds1∆* forks appear to retain Pol𝛼 but accumulate Cdc45 and Rad51 in unreplicated areas. We infer that potential “chicken foot” fork restart structures that are proposed in *cds1∆* after HU [55] are either a small proportion of the restarting fork population or do not involve Cdc45 regression into replicated areas.

### Fork window size iteration suggests normal and collapsing Rad51 and Cdc45 functions at replication forks

Current studies show that fork size may vary across strains and conditions, prompting us to ask whether fork complexes extend beyond the replicated tip into both replicated and unreplicated areas of BrdU tracts. A key feature of R-ODD-BLOBS is that users can define the fork window around tract tips, varying the number of pixels on either side to rigorously analyze the distribution and pattern of protein around each fork. Replicated and unreplicated sides of the fork can be defined individually. R-ODD-BLOBS summarizes colocalization percentages in a grouped bar graph for comparison. To assess the impact of potentially longer protein tracts on analysis, we incrementally expanded the fork window into neighboring replicated and unreplicated pixels (Fig. 5D and E).

Rad51 is a recombination protein that immunoprecipitates with replication fork components [56]. We had seen that most Rad51 was not directly detected at fork tips (Fig. S8B) and that Rad51 average lengths were similar (Fig. 5B) between all genotypes. We predicted that Rad51 filament formation for HR might extend from a broken fork into unreplicated chromatin; if so, increasing the fork window into adjacent unreplicated pixels could detect whether Rad51 spreads around a fork. By iterating the size of the fork in 2-px increments on each side of the tip (Fig. 5D and Fig. S9A), we found that most Rad51 in *mrc1∆* and wt is within 10 px (~3 kb) of a fork on the unreplicated side. The *cds1∆* forks have approximately 50% of Rad51 either fork-adjacent within 10 px, or in entirely unreplicated areas that are not fork-associated. As fork size expanded into adjacent replicated areas, there was less Rad51 with replicated signal and more at fork tips. We concluded that Rad51 is primarily fork-colocalized, particularly in wt and *mrc1∆* during HU restart, and that *cds1∆* forks have increased Rad51 in unreplicated areas (Fig. 5F and G).

Cdc45 is a part of the CMG helicase complex that unwinds dsDNA. Both *cds1∆* and *mrc1∆* cells accumulate large amounts of single-stranded DNA in HU, which is believed to be caused by continued CMG activity in the absence of replication arrest. Because Cdc45 distribution might also be affected by fork-window size, we iterated the number of pixels around each fork tip (Fig. 5E and Fig. S9B). Most Cdc45 was at the tips in wt and *mrc1∆* (55% to 70%), increasing to over 70% Cdc45 fork-associated with a 4 × 4 px window. The *cds1∆* forks showed that over 60% of Cdc45 was in unreplicated areas with a 2 × 2 px fork window. Increasing the fork window enriched Cdc45 at *cds1∆* forks to approximately 55%, with the remaining Cdc45 in unreplicated areas. We concluded that Cdc45 is mostly fork-associated in wt and *mrc1∆* cells (Fig. 5G), and Cdc45 distance might determine a range of healthy fork size during restart. In contrast, Cdc45 is found away from forks into unreplicated areas in *cds1∆* samples during HU recovery, consistent with uncoupled CMG, and fork structure loss even as DNA synthesis proceeds.

### DNA damage signal distribution is asymmetric at HU-restart forks

**Fig. 6.**
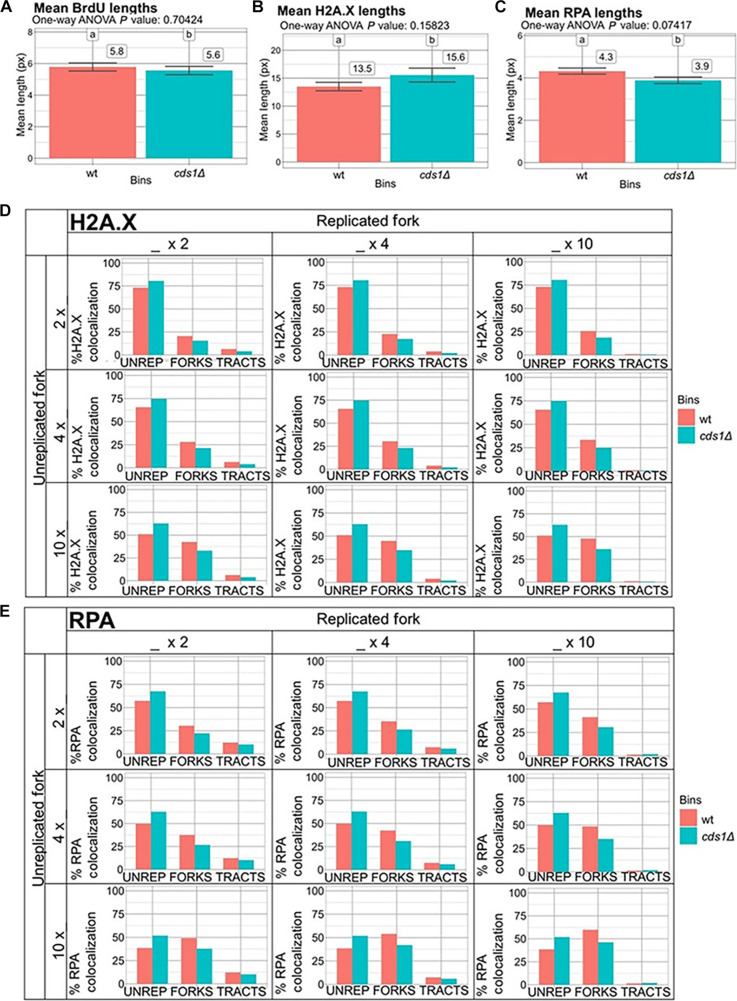
H2A.X and RPA distribution around replicated tract tips is largely unreplicated, with small differences between wild type and *cds1∆.* Thresholding and smoothing values determined from Figs. S9 and S10 were used to determine tract lengths of BrdU and the proteins H2A.X and RPA using R-ODD-BLOBS. Three replicates were used for each of the wt and *cds1Δ* cells. Cells were treated with 12 mM HU for 4 h, then released into fresh medium containing BrdU to detect BrdU incorporation after 30 min. All values are shown in pixels (px). The populations were compared using a 1-way ANOVA with Tukey’s HSD post-hoc correction. (A) Average BrdU-tracts post HU are of similar length in wt and *cds1∆*. There is no significant variation between genotypes (*P* < 0.704). (B) H2A.X protein tracts are similar in wt and *cds1∆* tracts after HU (*P* < 0.158), and are the longest average tracts found in our current study of different proteins. (C) Average RPA protein tract lengths are similar in wt and *cds1∆* (*P* < 0.07). (D) Analysis of H2A.X distribution around replicated tract tips shows that H2A.X is found mostly in unreplicated regions for both wt and *cds1∆*. As the number of unreplicated fork pixels increased (down), the absolute number of unreplicated H2A.X decreased and became FORK associated; however, the proportional difference between wt and *cds1∆* remained the same. As the number of replicated fork pixels increased (left to right), H2A.X detection was lost at TRACTS and had a small impact on the overall proportion of H2A.X located at FORKS. Generally, more H2A.X was at wt FORKS compared to more UNREP in *cds1∆.* (E) Analysis of RPA distribution around replicated tract tips. As the number of unreplicated fork pixels increased from 2 to 10 (down), RPA became FORK associated and wt had more RPA that was FORK associated than *cds1∆.* As the number of replicated fork pixels increased (2 px left to 10 px right), RPA was lost at TRACTS and became a part of FORKS. The 2 × 2 and 10 × 10 windows preserved the overall pattern of detection: more RPA is upstream of forks in *cds1∆,* while more RPA is at forks in wt, particularly with the 10 × 10 window.

To understand the differences between wt and *cds1∆* forks, we next examined DNA damage mark H2A.X and ssDNA binding protein RPA. After 4 h in HU, we labeled cells with BrdU and prepared fibers to acquire images of wt and *cds1∆* replicated tracts. Our optimized analysis was used to determine threshold values (Fig. S10; DNA, 100; BrdU, 300; H2A.X, 400; RPA, 300) and then smoothing values to ligate subresolution gaps in signal (Fig. S11; DNA, 3; BrdU, 3; H2A.X, 4; and RPA, 4). Consistent with the earlier Cdc45/Rad51 datasets, we found BrdU tract lengths of approximately ~1.95 kb (5.8 px) in wt and ~1.89 kb (5.6 px) in *cds1∆* (Fig. 6A).

We were interested in testing the chromatin mark H2A.X, which is considered an early mark of DNA damage signaling (Fig. 6B). We observed different levels of H2A.X signal at replicated regions, ranging from none to extensive (Figs. S12 and S13). At an induced DSB, H2A.X spreads for many kilobases on both sides around a DSB [57]. We found that H2A.X tract lengths in wt were 13.5 px (~4.54 kb) and 15.6 px (~5.26 kb) in *cds1∆* (Fig. 6B). This is consistent with H2A.X spread around DNA damage that occurs during HU arrest and/or recovery. RPA is also believed to form filaments that extend in areas of ssDNA, and we saw similar patterns of more or less RPA around replicated tracts (Fig. S13). Because RPA accumulates in *cds1∆* nuclei during HU and restart [23], we expected that RPA tracts would be longer and more frequent at *cds1∆* forks. Unexpectedly, we found similar RPA lengths in wt (4.3 px,~1.45 kb) and *cds1∆* (3.9 px, ~1.31 kb) (Fig. 6C).

To test whether H2A.X and RPA distributions were different in wt and *cds1∆*, we again iterated the fork window size around BrdU-tract tips. H2A.X decorates unrepaired DSB and replication stress [24], particularly in replication checkpoint mutants such as *cds1∆* [23]. We expected H2A.X to localize at fork-tip areas during restart; instead, we found that most H2A.X was in unreplicated areas (Fig. 6D). Increasing the unreplicated side of fork windows had a dominant effect, indicating that ~40% of wt or ~30% of *cds1∆* H2A.X is near a fork tip into unreplicated chromatin. The majority of H2A.X is in unreplicated DNA more than 10 px (~3.3 kb) away, particularly in *cds1∆.* This asymmetric H2A.X spread may reflect damaged forks that cannot restart and do not label with BrdU, and reduced H2A.X decoration on newly replicated chromatin within 30 min of restart.

RPA, like Rad51, forms filaments around ssDNA during DNA replication and damage resection [45]. ssDNA is produced at stalled replication forks through different methods, including residual helicase activity [4,13,19,58], resection [20,59,60], recombination [61,62], or fork regression [63,64]. We expected to find RPA associated with fork tips in all cases; however, we saw most RPA in unreplicated areas of both wt and *cds1∆* (Fig. 6E). Increasing unreplicated pixels at the fork window shifted RPA association to fork tips in wt (4 to 10 px, Fig. 6E), while *cds1∆* still had most RPA in unreplicated areas. Consistent with our finding that Cdc45 is displaced into unreplicated DNA away from *cds1∆* forks (Fig. 5E), our RPA analysis indicates that *cds1∆* forks have a higher risk of CMG uncoupling and ssDNA accumulation in unreplicated DNA.

### Combined protein distribution analysis predicts fork anatomy during HU restart

**Fig. 7.**
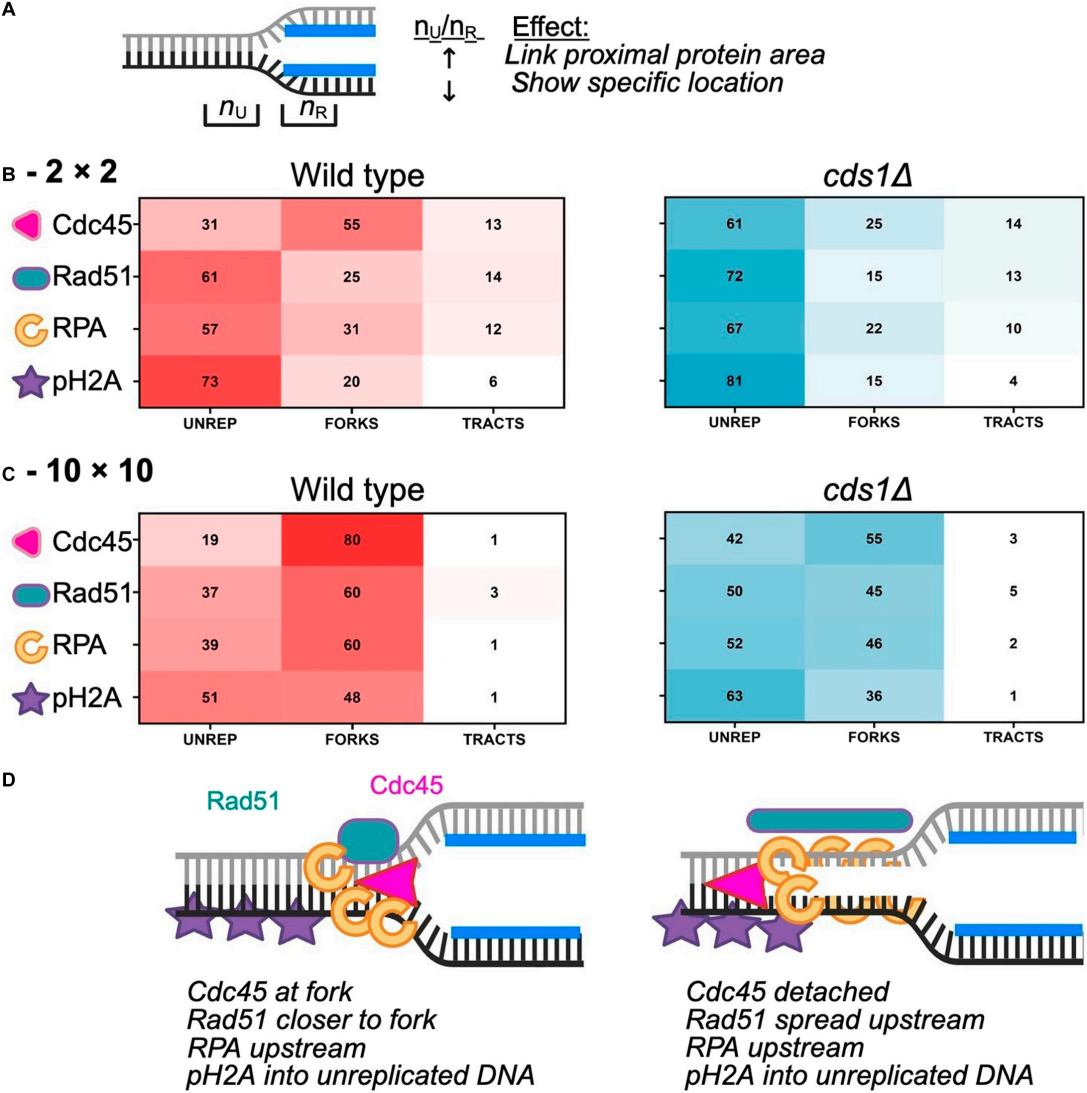
Cdc45, Rad51, H2A.X, and RPA colocalization comparison suggests altered fork structure in *cds1*∆. The colocalization proportion of multiple proteins can be compared to identify a probable fork structure based on the likelihood of the protein being within the region. (A) The number of pixels included in the fork in either the unreplicated (*n*_U_) or replicated (*n*_R_) areas; R-ODD-BLOBS can test protein colocalization patterns. We test a small area around the fork: 2 × 2 px [shown in (B)]. The unreplicated (*n*_U_) = 2 px, replicated (*n*_R_) = 2 px, and the tip (1 px). The area considered is 5 px, covering ~1.7 kb. Similar to Figs. 5 and 6, we can also test a larger fork of 10 × 10 px [shown in (C)], spanning 21 px or ~7.0 kb; this shows the proximity of the immediate fork and how close proteins are to the fork. (B) The proportion of Cdc45, Rad51, RPA, and pH2A in the unreplicated (UNREP), fork (FORK), and replicated (REP) regions of wt and *cds1∆* within 2 px from the fork (2 × 2 px) was compiled into a heatmap. The saturated, darker color represents a larger proportion of colocalization. In wt, Rad51, RPA, and pH2A are likely to colocalize to unreplicated regions, while Cdc45 is more likely to colocalize to the fork. In *cds1∆,* all 4 proteins are more likely to be found at the unreplicated region. (C) The proportion of Cdc45, Rad51, RPA, and pH2A in the unreplicated (UNREP), fork (FORK), and replicated (TRACTS) regions of wt and *cds1∆* within 10 px from the fork (10 × 10 px) was compiled into a heatmap. The saturated, darker color represents a larger proportion of colocalization. In wt, Cdc45, Rad51, and RPA were within the fork region. While pH2A is almost evenly divided between the unreplicated and fork region. In *cds1∆,* Cdc45, Rad51, and RPA are distributed around 50% in the unreplicated region and 50% at forks. pH2A still has a slight preference toward unreplicated regions. (D) “Cartoon” fork models that compare wt to the *cds1∆,* showing the difference in probable protein colocalization. Cdc45 in *cds1∆* was shifted from the tip and into the unreplicated region, invoking ssDNA that is unreplicated.

The *cds1∆* forks are unstable after 4 h HU, even though synthesis proceeds; *cds1∆* cells show ~1% viability, and bulk analysis shows extensive DNA damage and ssDNA [23]. To understand whether replication protein distribution is altered in *cds1∆* compared to wt, we compared the distribution of Cdc45, Rad51, RPA, and H2A.X in unreplicated, fork, and replicated areas (Fig. 7 and Fig. S13). When the number of unreplicated pixels (*n*_U_) or replicated pixels (*n*_R_) is increased in R-ODD-BLOBS, the effect will show if a protein is proximal to forks. Decreasing *n*_U_ and *n*_R_ will show fork decoration constrained to tips. We compared region distributions of all proteins in wt compared to *cds1∆* with a constrained (2 × 2 px) or expansive (10 × 10 px) fork window (Fig. S13). In wt, the proteins Rad51, RPA, and H2A.X were unreplicated-adjacent in a narrow 2 × 2 window and became more fork associated using a 10 × 10 window (Fig. 7B and C). Cdc45 in wt was mostly fork tip associated regardless of window size. In contrast, Rad51, Cdc45, RPA, and H2A.X were more unreplicated with a 2 × 2 window, but 30% to 50% of each protein was fork tip adjacent in a 10 × 10 window (Fig. 7B and C). Less protein associated with fork tips in *cds1∆* shows that the *cds1∆* fork population is distinctive and different from wt during HU recovery. The *cds1∆* forks show Cdc45 detachment, and RPA, Rad51, and H2A.X damage and repair signals into unreplicated chromatin.

### Using R-ODD-BLOBS to reduce user variability and biases

Chromatin fiber data are rich and complex; manual analysis without rigorous parameterization can introduce evaluator biases, leading to different interpretations among evaluators, creating uncertainty and inconclusive results on the nature of replication fork collapse. Other methods to analyze chromatin fiber or combing data have been successful, some within a commercial context. For example, Bionano Saphyr maps spatially resolved origin firing across the genome on spread fibers [45]. Genomic Vision’s FiberStudio can identify the length of synthesized tracts, as well as origin, termination, or ongoing synthesis [43]. Both methods assess synthesis patterns using spatial patterns of CldU/IdU-labeled replication tracts. R-ODD-BLOBS can detect tracts and forks in addition to replication proteins, adding a unique complement to label-only methods.

R-ODD-BLOBS addressed evaluator bias by processing fiber images into binary datasets. Exploring threshold and smoothing parameters and iteratively adjusting the fork window sides promote consistent analysis across multiple users. Parameter analysis follows the principle of exploring the parameter’s range to determine how it affects the data. After viewing how thresholding affected the data (Fig. 4 and Figs. S1, S2, and S10), we tested a “preliminary baseline threshold”, which could be assessed relative to fiber images (i.e., Fig. S12) and identify signal outliers that could skew iterative tests. Next, in iterative thresholding analysis (Fig. 4 and Figs. S10 and S11), we used a 0 to 1,000 fluorescent intensity range at increments of 100; this allowed us to determine how tract lengths changed relative to threshold (Fig. 3). By identifying each threshold value that stabilizes tract length, avoid including nonspecific signals while retaining all “real” data. We suggest that the best thresholding value is lower than the plateau of average length. However, other users and analyses may require different threshold points for different datasets; for example, the point of inflection could be used as the point between the highest and lowest average lengths for each channel. In our data, midpoint thresholds would be 100 for DNA, and 200 for BrdU, Rad51, and Cdc45.

As thresholding and smoothing analysis allow users to rationalize the values they select, there is a need for easy access to and low-commitment methods for exploring and investigating the effects of thresholding and smoothing. Figure 2 suggests guidelines for data processing and analysis, and reasoning behind the decisions that were made. Regardless of the method chosen, by specifying the threshold selection method and values, R-ODD-BLOBS promotes replication of our analysis and full disclosure of methods used. Similarly, smoothing analysis allows ligation of small gaps in the signal, which are caused by labeling inconsistency or other experimental variation. Because 1- to 3-px gaps are below the Abbé limit of resolution in our system, analyses with and without smoothing may help to understand tracts that appear continuous by eye but discontinuous after thresholding (i.e., Fig. S12). Users with super-resolution capability will have different detection limits in their images based on pixel size and system.

## Conclusion

We present R-ODD-BLOBS as a high-resolution method for replication fork patterning and discovery from chromatin fiber images. Our R-based code has improved the computational stability and speed of ODD-BLOBS linear data analysis [36], allowing iterative testing to optimize thresholding and smoothing parameters. Thresholding is important to discriminate true signal from background, and describe rigorously across datasets. The “Smooth It” function differentiates biological signal gaps from stochastic noise. We found that smoothing parameters suggested by the Abbé resolution limit (3 to 4 px depending on wavelength) preserve biological patterns to be closer to visual analysis, but prevent artificial merging of distinct tracts. Smoothing in R-ODD-BLOBS occurs quickly to allow the user to test the impact of gap closing on fork protein association. However, alternative methods of joining signals have been successful at mathematically assessing the impact of connecting signal gaps on replicated tracts [42–45]; these methods will be tested and implemented into future versions of R-ODD-BLOBS fork window analysis. Our iterative refinement of fork window zones around the replicated tips of each tract provides rigid spatial frameworks to map replication protein mapping at wt and mutant forks.

### Structural uncoupling and protein spread during HU restart depend on checkpoint competency

Our computational analysis reveals distinctive structural differences in the replication stress mutant *cds1∆.* We observed displacement of Cdc45 into unreplicated regions, agreeing with earlier evidence that Cds1 protein helps connect the CMG helicase for stability during HU block and release. Cdc45 is part of the CMG complex with Mcm2-7 and GINS, which unwinds dsDNA during replication [47]. Cdc45 uncoupling is consistent with more RPA and Rad51 in unreplicated areas near fork tips, and with ssDNA accumulation into unreplicated areas far from forks. While our aggregate data of Cdc45 around the fork window is consistent with biochemical and sequencing analysis of Cds1 effects, Cdc45 could also appear at unreplicated regions during origin firing [65] or after prereplication complexes are destroyed [66]. Together, our analysis provides a framework for next steps to model individual fork structures that probe the differences between wt, which survives HU, and *mrc1∆* or *cds1∆,* which die in HU.

R-ODD-BLOBS has detected and described spatial patterns for proteins that may form tracts or filaments, such as RPA, H2A.X, and Rad51. RPA, like Rad51, stabilizes ssDNA and can form filaments [67]. We found that these proteins show similar spread into unreplicated areas of fibers. Some instances can be connected to an expanded fork window, consistent with the formation of repair protein filaments for HR repair or fork restart. The similarity in RPA and H2A.X patterns of *cds1Δ* and wt suggests that lethal levels of DNA damage have not occurred within 30 min after HU release. In turn, *cds1∆* deregulated late origin firing and excess ssDNA [23] could become cell-killing DNA damage in the next cell cycles [68].

Together, these colocalization datasets across multiple fiber experiments and images quantify structural trends and provide an alternative to manual assessment of fork collapse from fiber images.

### Asymmetric H2A.X and the fork as a barrier to signal spread

Mapping the DNA double-strand break marker H2A.X [24,27] around fork tips revealed an unexpected finding: the majority of H2A.X signal is in unreplicated areas and is not symmetric around tract tips. Conceptually, fork collapse in *cds1∆* and *mrc1∆* suggests that fork complexes are unstable, causing DNA breaks at some point during HU or after restart. The nature of collapse and the variation between mutants or forks within a population are largely unknown. We predicted that fork collapse in *cds1∆* would be at replicated tips, and that we would find H2A.X at forks. Formally, end resection at a collapsed fork might remove BrdU signal in an area with H2A.X. DNA damage from other sources, such as reactive oxygen species, could cause DSBs in nonreplicated areas [24,69].

Yet, our finding that H2A.X, RPA, Rad51, and Cdc45 are all increased in *cds1∆* unreplicated areas suggests that the replication fork may be a structural barrier to damage signal spread. For example, H2A.X may not be present in the newly replicated DNA, or the fork complex might obstruct H2A.X phosphorylation. H2A.X asymmetry can also be influenced by topologically associated chromatin domains, cohesin complexes, or transcription [25,70]. Since most DNA repair studies describe symmetric H2A.X phosphorylation, fork asymmetry provides another example for context- and structure-dependent recognition of DNA repair factors.

Because R-ODD-BLOBS can deconvolve complex phenotypes, our future work will correlate single-fork protein patterns with local genomic context. Our fork analysis to date has reinforced the similar phenotypes but different roles of Cds1 and Mrc1 in genome stability [49,71,72]. Multicolor labeling for replication restart, and FISH of chromosome fragile sites will develop our modeling to explore how epigenetic factors and structural barriers influence genome stability at single replication forks.

## Data Availability

As described in Table 2, R-ODD-BLOBS code is free and publicly available, and can be accessed through https://github.com/SabatinosLab/R-ODD-BLOBS. Data supporting the reported results are available upon request to S.S. (ssabatinos@torontomu.ca).
