# Supplementary material for: Analyzing DNA Replication Fork Stability and Collapse Using Chromatin Fiber Analysis and the R-ODD-BLOBS Program
Source: Comput Struct Biotechnol J. 2026 Jul 22;35(1):0140. doi: 10.34133/csbj.0140 (PMC13389046; doi:10.34133/csbj.0140)
Supplement: Supplementary 1 — Figs. S1 to S13 [file csbj.0140.f1.docx]

*
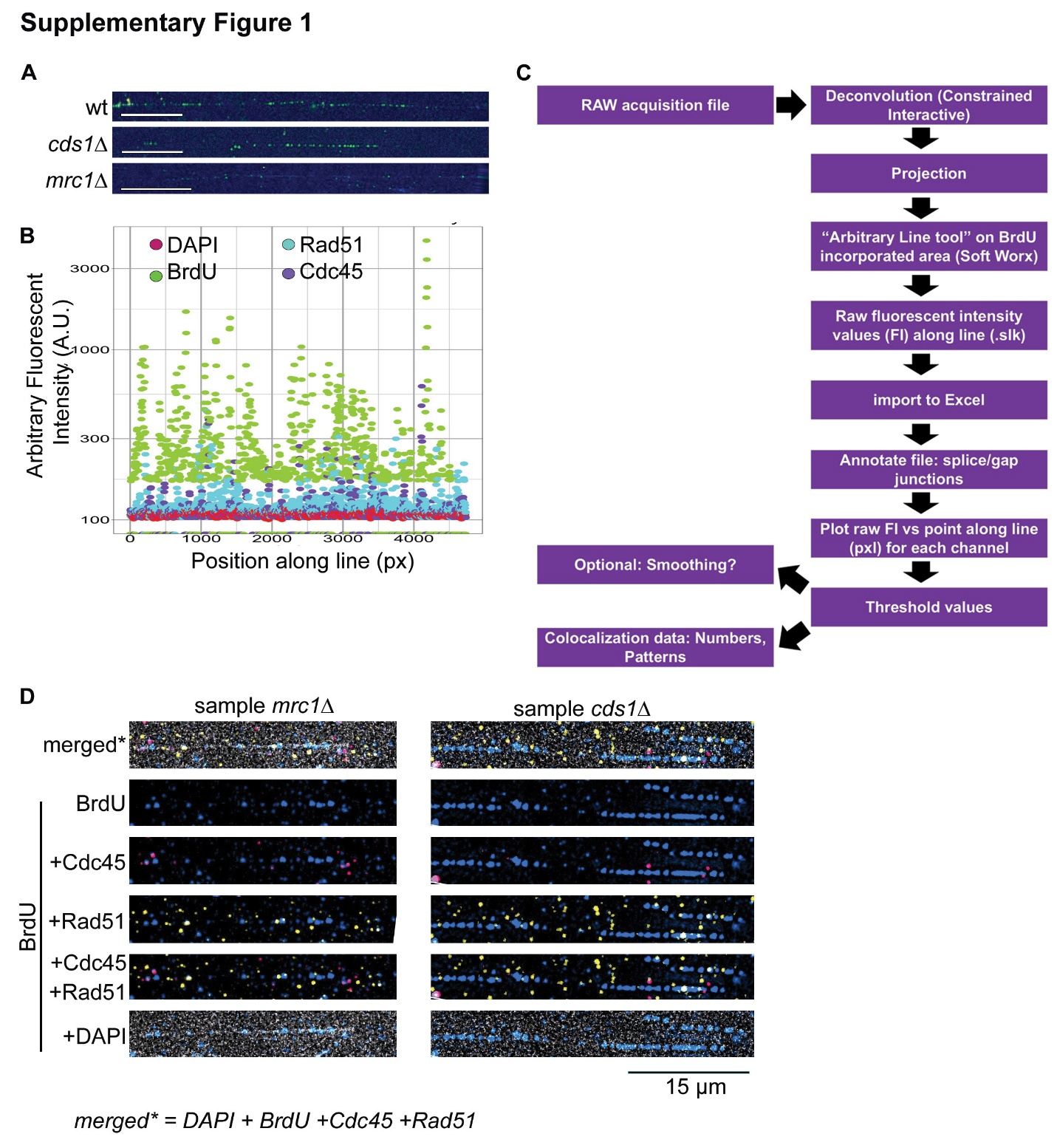
*

**Supplementary Figure 1:** **Linear data acquired from chromatin fibers.** Chromatin fibers stained for DNA (DAPI), BrdU, Cdc45 and Rad51 were prepared, and images of fibers were traced along the fiber line. Fluorescent intensity values were imported into spreadsheets for uploading into R-ODD-BLOBS.

(A) Sample images of wt, *cds1∆,* and *mrc1∆* fibers that were blocked for 4h in HU, and released for 30 min in the presence of BrdU. Fibers show BrdU (green) and DAPI detection. Scale bars 15 µm.

(B) Scatter plot of initial signal analysis in R-ODD-BLOBS. Pixels along the line (x-axis) show plotted arbitrary fluorescent intensity values (AU, y-axis; log10 scale) for DNA (DAPI, red), BrdU (green), Rad51 (cyan), and purple (Cdc45). This initial scatter plot suggests an initial ‘baseline’ threshold and data outliers. For example, BrdU (green) has a potential lower limit of 150, above which the signal is more likely to describe BrdU incorporation.

(C) Flow chart describing steps in R-ODD-BLOBS analysis. Microscope images (RAW files) are deconvolved and projected, and the fluorescent data along lines is recorded for all channels (BrdU and proteins) using a line drawing tool (Arbitrary Line Tool in SoftWoRX, or similar program). Fluorescent intensities at each pixel and pixel position information are imported to a spreadsheet (i.e. Excel), and annotated to specify breaks in signal. Multiple fiber traces across images can be concatenated or can be pooled at a later stage; we recommend for a more robust analysis that multiple images be processed together at least once to understand staining/labeling variation between images and experimental replicates (eg. Supplemental Figure S2). The raw fluorescent intensity is plotted for each pixel along a line (as in B), and a preliminary threshold can be assigned. A full, iterative threshold analysis can be performed as described for Figures 2A and 3. Small sub-resolution gaps in signal can be tested for their impact on results using Smooth-It, as described in Figures 2B and 4. Finally, co-localization of signals with replicated tract ends and between replication proteins can be assessed by examining above-threshold signals (length, frequency, distribution, *etc*).

(D) Sample *mrc1∆* and *cds1∆* fibers labeled with BrdU (blue), Rad51 (yellow), Cdc45 (pink), and DAPI. Data is acquired along a line following the DAPI/DNA stain to determine proteins associated with un-replicated areas.


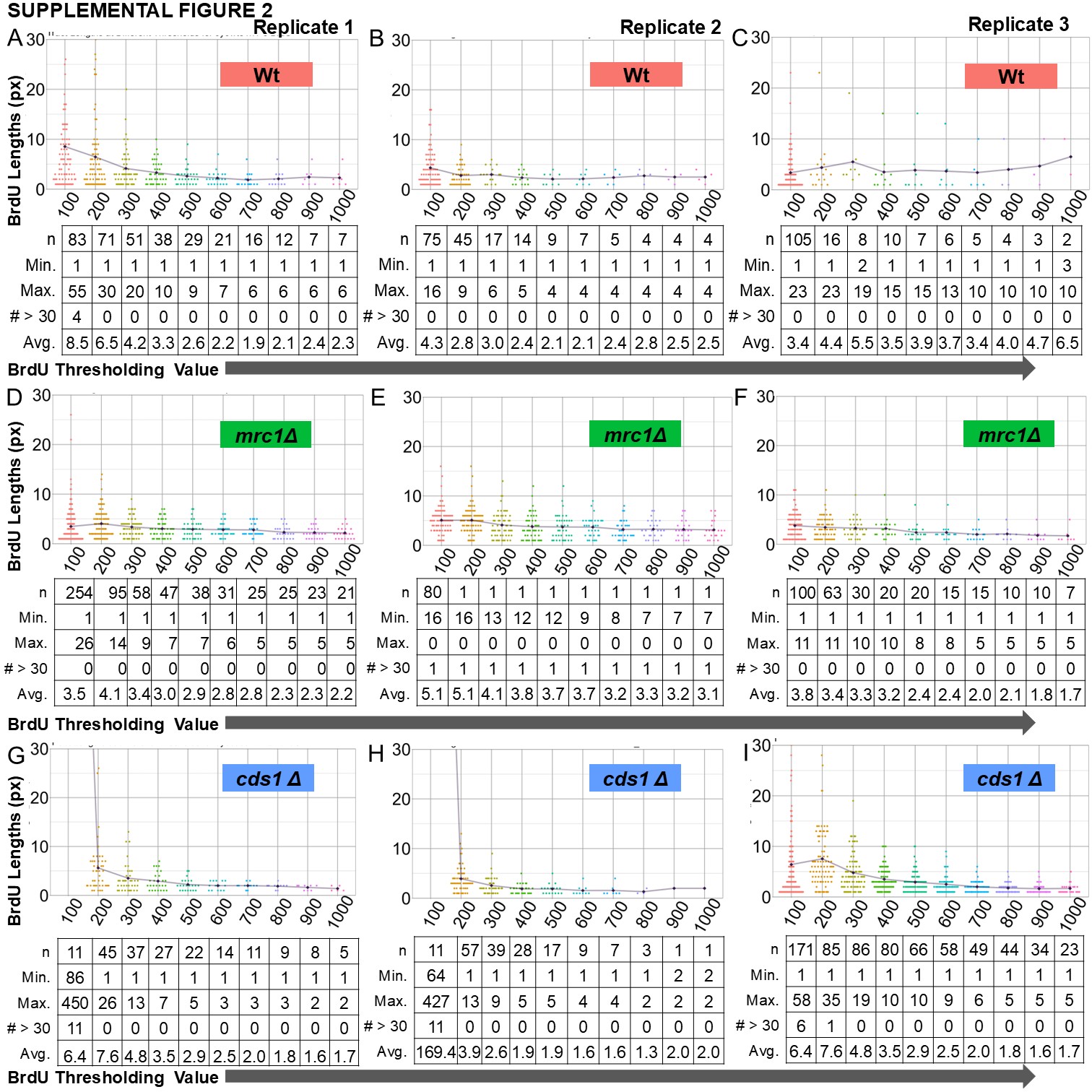


**Supplementary Figure 2: Replicates of BrdU thresholding in wt, *cds1Δ* and *mrc1Δ* suggest that a common threshold exists across multiple experiments/datasets.** Line-traced chromatin fiber data were processed using R-ODD-BLOBS using different thresholds for each channel. Thresholds ranging from 100 to 1000 were used to test the effect of higher thresholds on the tract length of DNA synthesis (BrdU) in wt, *mrc1Δ,* and *cds1Δ*. Each channel was iterated separately. A baseline “placeholder” threshold was used for each channel not tested. Baseline thresholds were calculated from a scatter plot of intensities (shown in Supplemental Figure S1). (A-C) Bee swarm plots of newly synthesized tracts (BrdU) in wt from 100- 1000. (D-F) Bee swarm plots of newly synthesized tracts (BrdU) in *mrc1Δ* from 100- 1000. (G-I) Bee swarm plots of newly synthesized tracts (BrdU) in *cds1Δ* from 100- 1000.


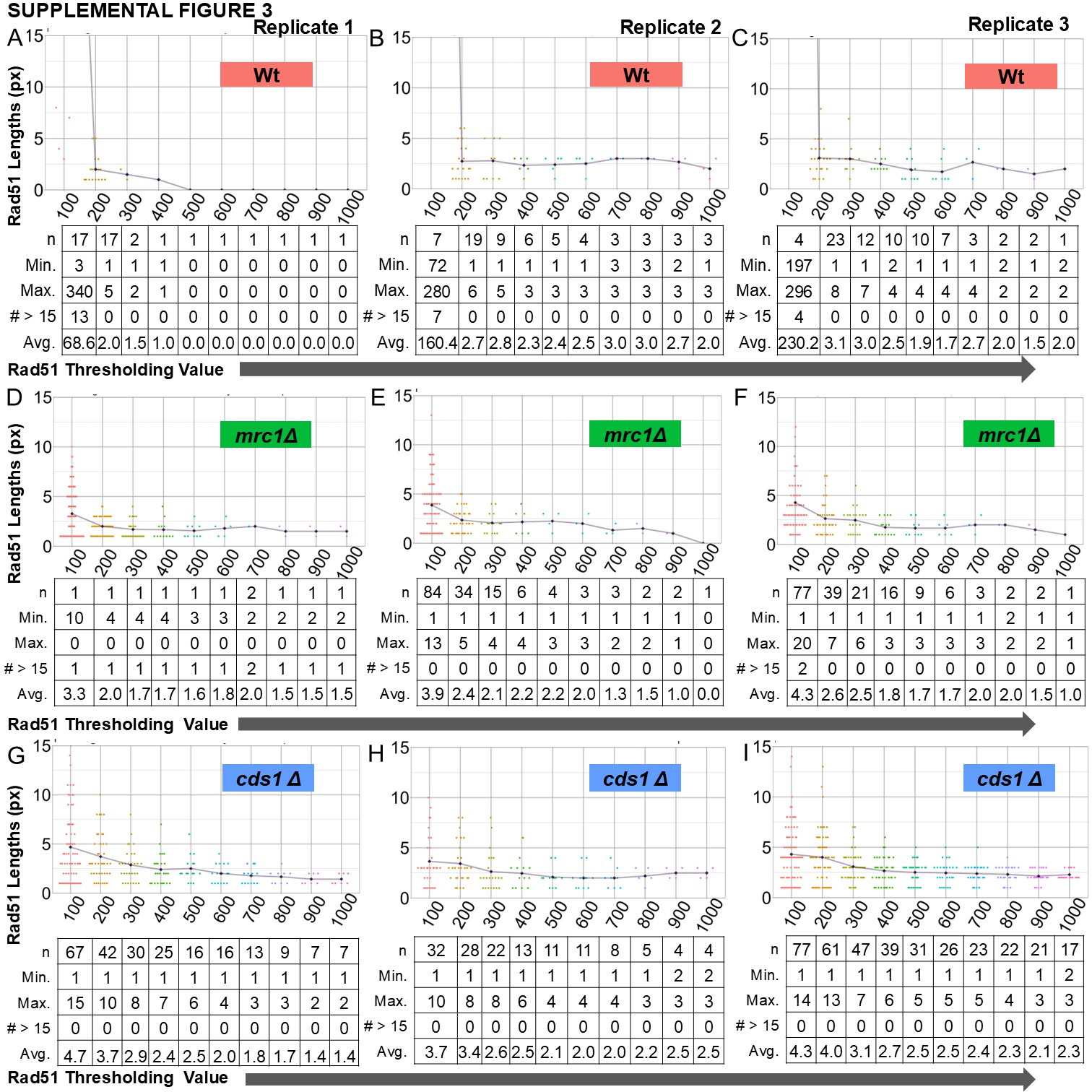


**Supplementary Figure 3: Individual replicates of Rad51 thresholding for wt, *cds1Δ* and *mrc1Δ*.** The intensities of the line-traced chromatin fiber data were processed using R-ODD-BLOBS using different thresholds for each channel. Thresholds from 100 to 1000 were used to test the effect of higher thresholds on Rad51 protein length in wt, *mrc1Δ,* and *cds1Δ*. Each channel was iterated separately. A baseline “placeholder” threshold was used for each channel not tested. Baseline thresholds were calculated from a scatter plot of intensities (shown in Supplemental Figure S1). (A-C) Bee swarm plots of Rad51 lengths in wt from 100- 1000. (D-F) Bee swarm plots of Rad51 lengths in *mrc1Δ* from 100- 1000. (G-I) Bee swarm plots of Rad51 lengths in *cds1Δ* from 100- 1000.


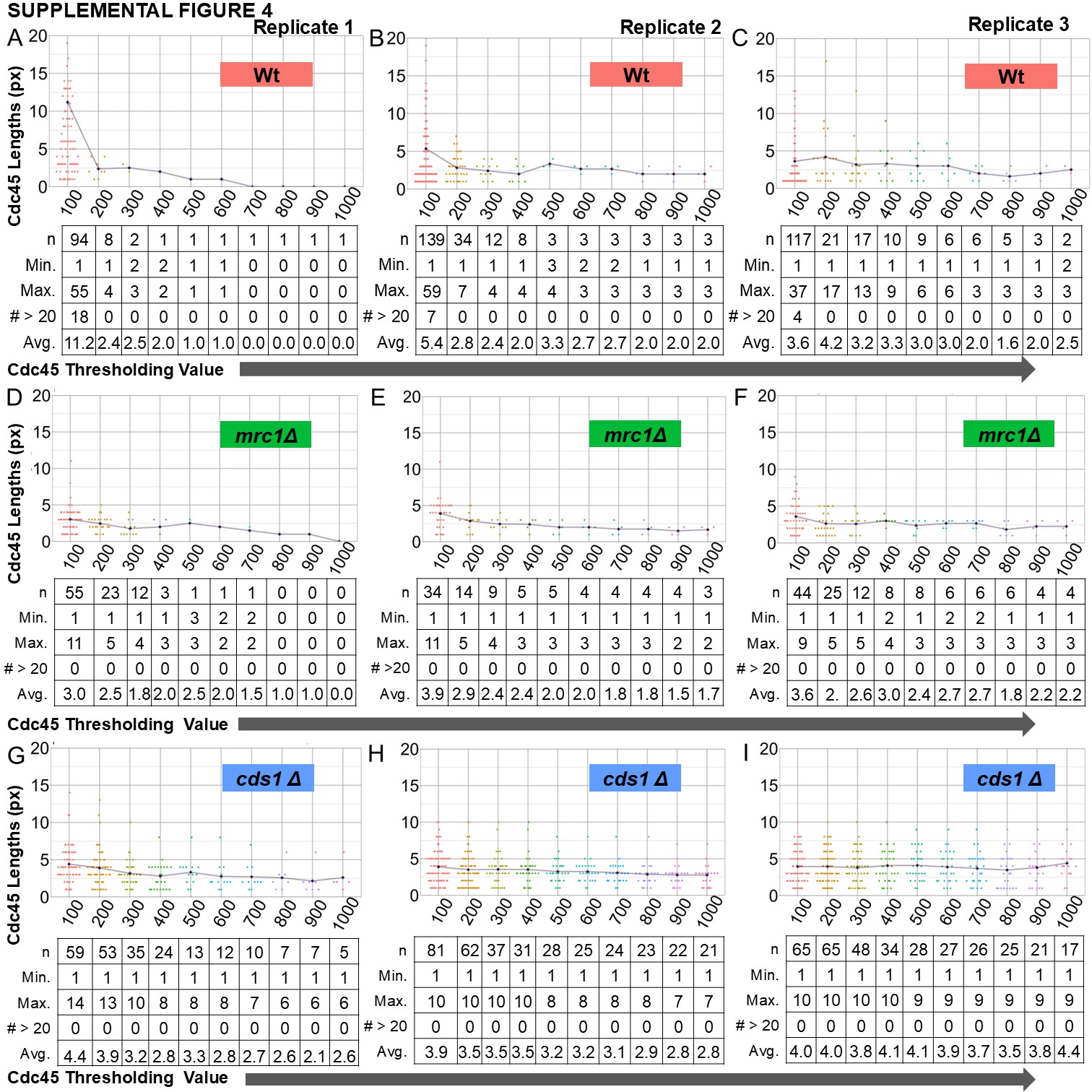


**Supplementary Figure 4:** **Individual replicates of Cdc45 thresholding for wt, *cds1Δ* and *mrc1Δ*.** The intensities of the line-traced chromatin fiber data were processed using R-ODD-BLOBS using different thresholds for each channel. Thresholds ranging from 100 to 1000 were used to test the effect of higher thresholds on Cdc45 protein length in wt, *mrc1Δ,* and *cds1Δ*. Each channel was iterated separately. A baseline “placeholder” threshold was used for each channel not tested. Baseline thresholds were calculated from a scatter plot of intensities (shown in Supplemental Figure S1). (A-C) Bee swarm plots of Cdc45 lengths in wt from 100- 1000. (D-F) Bee swarm plots of Cdc45 lengths in *mrc1Δ* from 100- 1000. (G-I) Bee swarm plots of Cdc45 lengths in *cds1Δ* from 100- 1000.


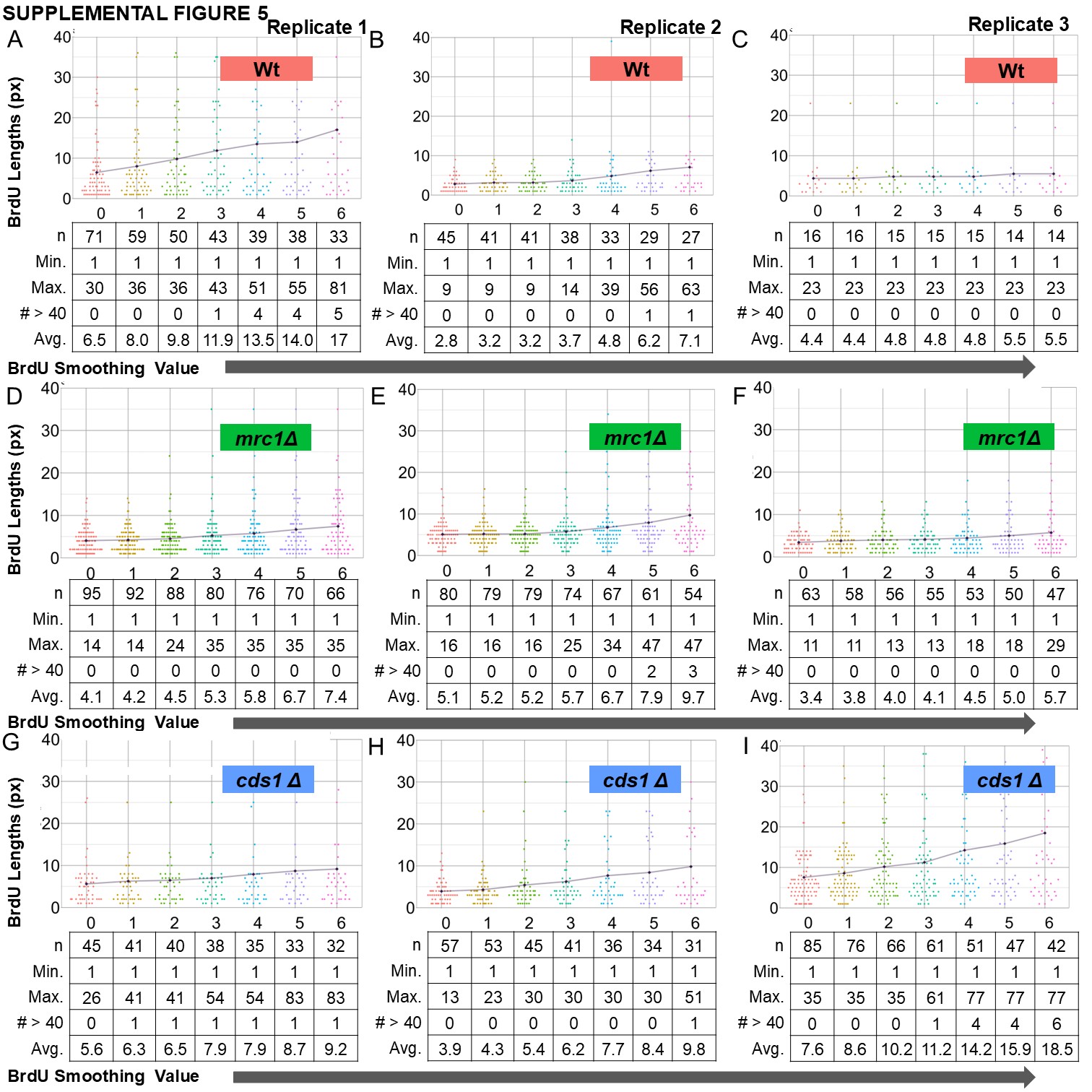


**Supplementary Figure 5:** **Individual replicates of BrdU smoothing for wt, *cds1Δ* and *mrc1Δ*.** The intensity of the line-traced chromatin fiber data was processed using R-ODD-BLOBS using previously determined threshold values: 100 for DNA; 200 for BrdU, Rad51, and Cdc45. Smoothing links tracts together if the number of pixels is equal to or less than the “smoothing value” used. (A-C) Bee swarm plots of the tract lengths in BrdU in wt at smoothing values 0 px to 6 px. (D-F) Bee swarm plots of the tract lengths in BrdU in *mrc1Δ* at smoothing values 0 px to 6 px. (G-I) Bee swarm plots of the BrdU tract lengths in *cds1Δ* at smoothing values 0 px to 6 px.

**
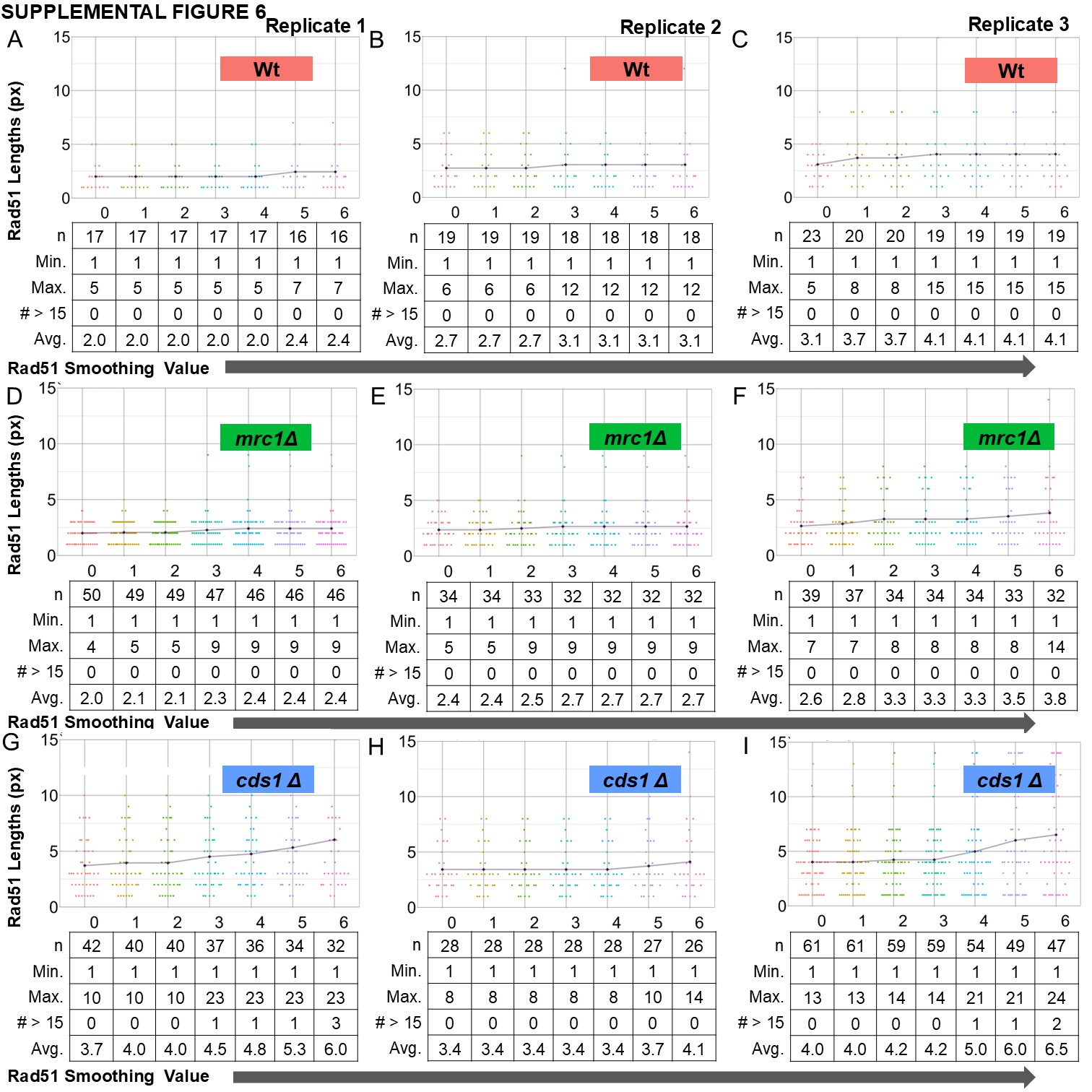
**

**Supplementary Figure 6: Individual replicates of Rad51 smoothing for wt, *cds1Δ* and *mrc1Δ*.** The intensity of the line-traced chromatin fiber data was processed using R-ODD-BLOBS using previously determined threshold values: 100 for DNA; 200 for BrdU, Rad51, and Cdc45. Smoothing links tracts together if the number of pixels is equal to or less than the “smoothing value” used. (A-C) Bee swarm plots of Rad51 protein lengths in wt at smoothing values 0 px to 6 px. (D-F) Bee swarm plots of Rad51 protein lengths in *mrc1Δ* at smoothing values 0 px to 6 px. (G-I) Bee swarm plots of Rad51 protein lengths in *cds1Δ* at smoothing values 0 px to 6 px.

**
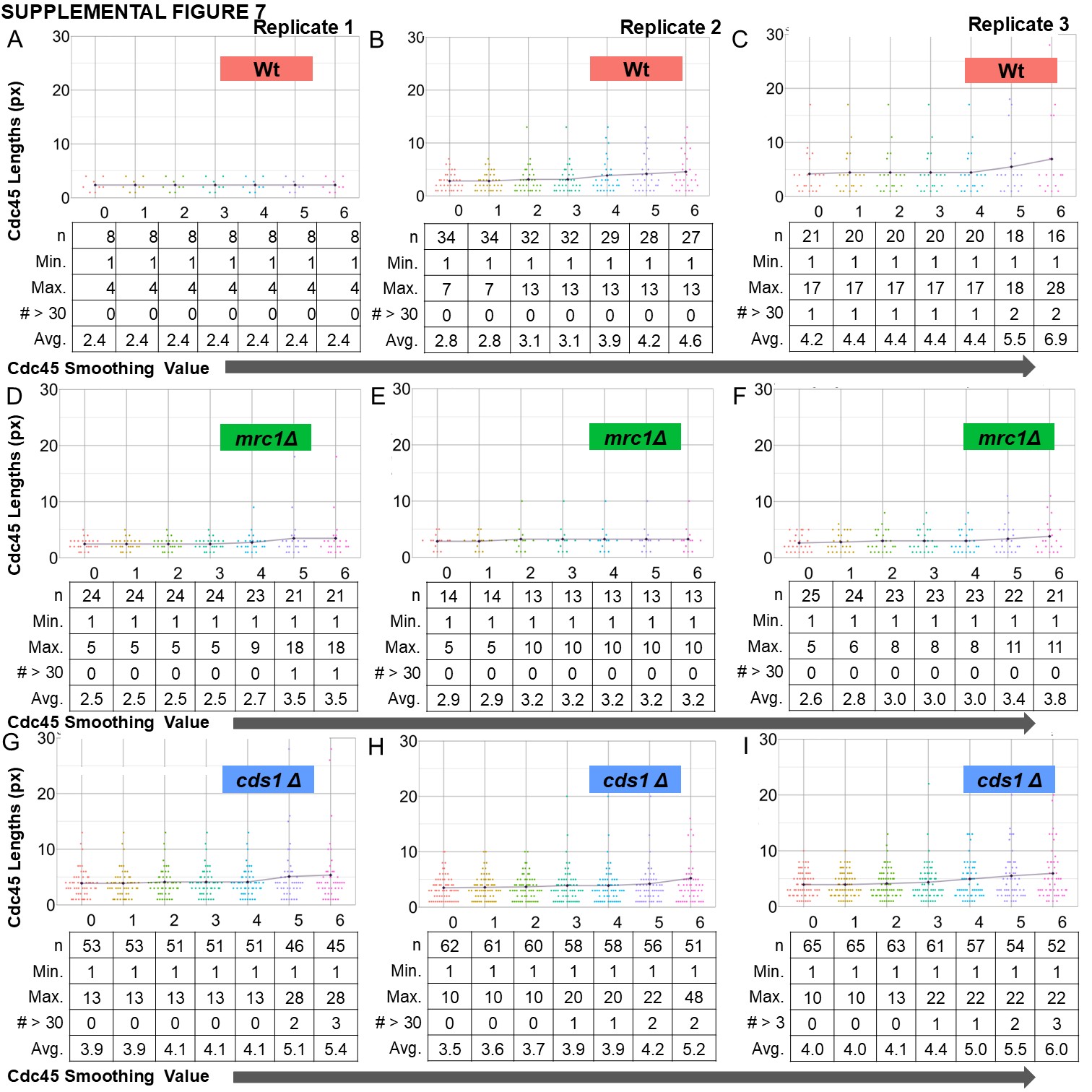
**

**Supplementary Figure 7: Individual replicates of Cdc45 smoothing iteration for wt, *cds1Δ* and *mrc1Δ*.** The intensity of the line-traced chromatin fiber data was processed using R-ODD-BLOBS using previously determined threshold values: 100 for DNA; 200 for BrdU, Rad51, and Cdc45. Smoothing links tracts together if the number of pixels is equal to or less than the “smoothing value” used. (A-C) Bee swarm plots of Cdc45 protein lengths in wt at smoothing values 0 px to 6 px. (D-F) Bee swarm plots of Cdc45 protein lengths in *mrc1Δ* at smoothing values 0 px to 6 px. (G-I) Bee swarm plots of Cdc45 protein lengths in *cds1Δ* at smoothing values 0 px to 6 px.


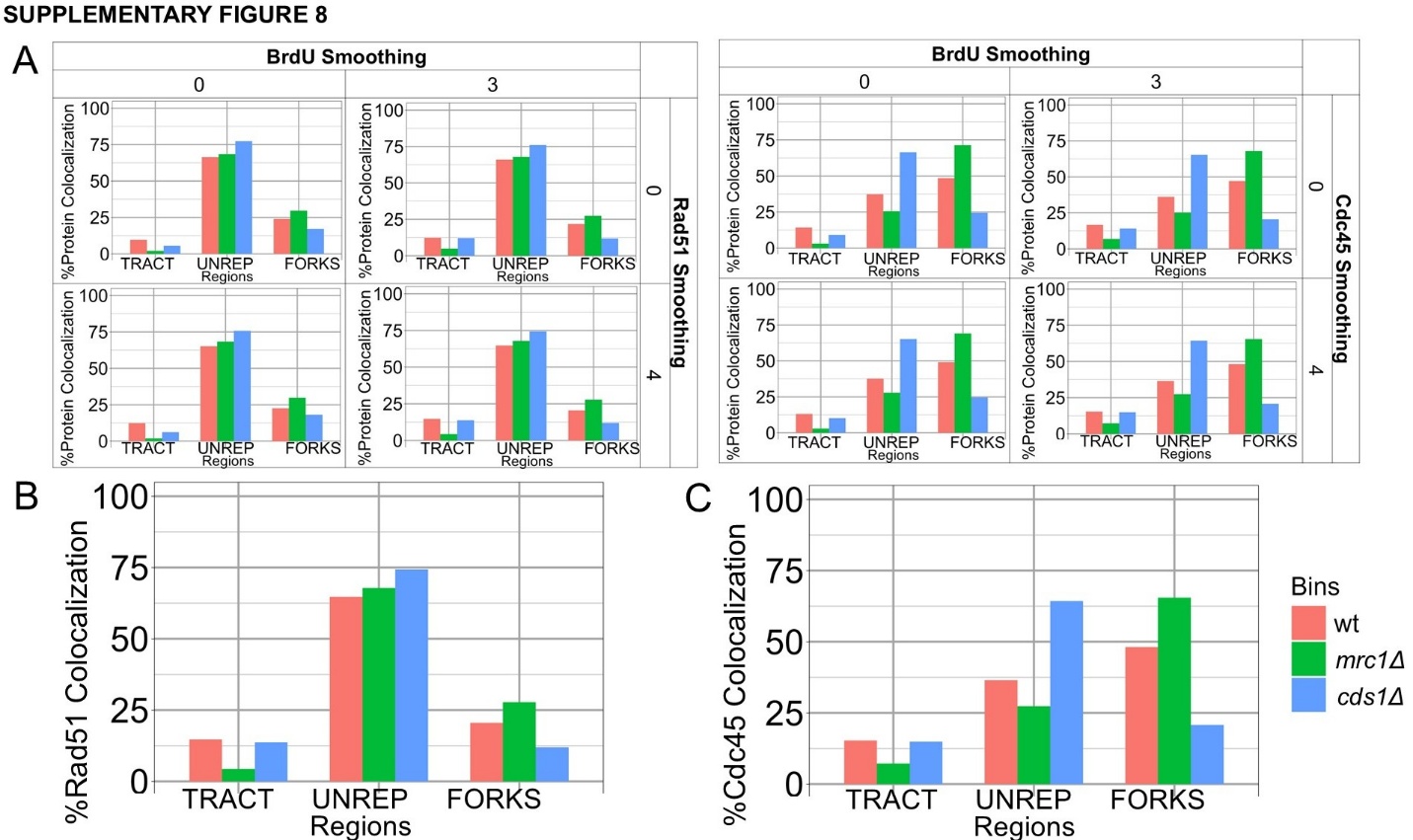


**Supplementary Figure 8: BrdU and protein smoothing parameters affect protein colocalization around replicated tracts.** The impact of smoothing on both BrdU and/or protein (Rad51, Cdc45) was compared to each other. Full iterations of smoothing parameters are shown in Supplementary Figures. Smoothing of 0 px or 3 px was used for BrdU, compared to smoothing of 0 px or 4 px for Rad51 and Cdc45. (A) Comparison of no-smoothing and smoothing on Rad51 distribution around replicated tract ends. BrdU-replicated tract tips are “FORKS”, and coincident with other BrdU areas is “TRACT”. Non-BrdU coincident with Rad51 is “UNREP” in the unreplicated areas, predicted to be remote from replication forks. (B) After optimal smoothing and thresholding are applied, a majority of Rad51 is in unreplicated areas for all genotypes. (C) Most Cdc45 is in unreplicated areas for *mrc1∆ (*green) *but* is fork-colocalization for *cds1∆ (blue)* and wt (pink).

**
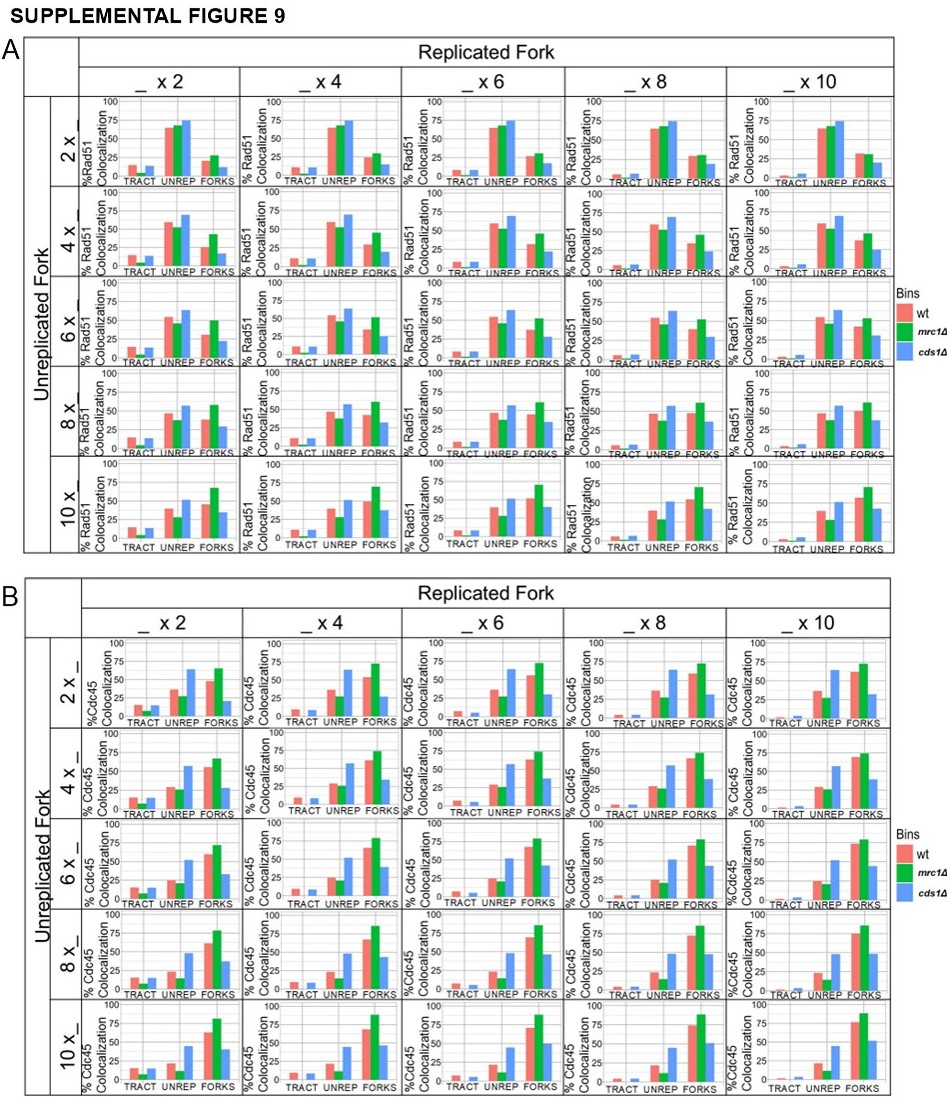
**

**Supplementary Figure 9: The effect of iterative window sizing on Rad51 and Cdc45 distribution coincident to forks.**

(A) Rad51 located with forks increases most in *mrc1∆*, suggesting that *mrc1∆* forks accumulate Rad51 that spreads from the fork into unreplicated areas. In contrast, *cds1∆* forks retain the most Rad51 in unreplicated areas that are away from forks, suggesting DNA damage and more spread out away from collapsing forks. From left to right, the number of pixels in the replicated fork area increases from 2 to 10. Extending the number of fork-pixels into the replicated area has a small effect on Rad51 localization, regardless of genotype. The 10x10 extreme situation (bottom right) is dominated by the effect of unreplicated pixels made fork proximal.

(B) Expanded window-size effects on Cdc45 colocalization for *cds1∆ (blue), mrc1∆ (green),* and wt (pink). With no pixels included on either side of the tip, Cdc45 is primarily unreplicated (“COLO UNR REGION”) regardless of the fork window around BrdU tips. As the grid goes from top to bottom, the number of pixels extending in the unreplicated area proximal to the fork increases from 2 to 10.


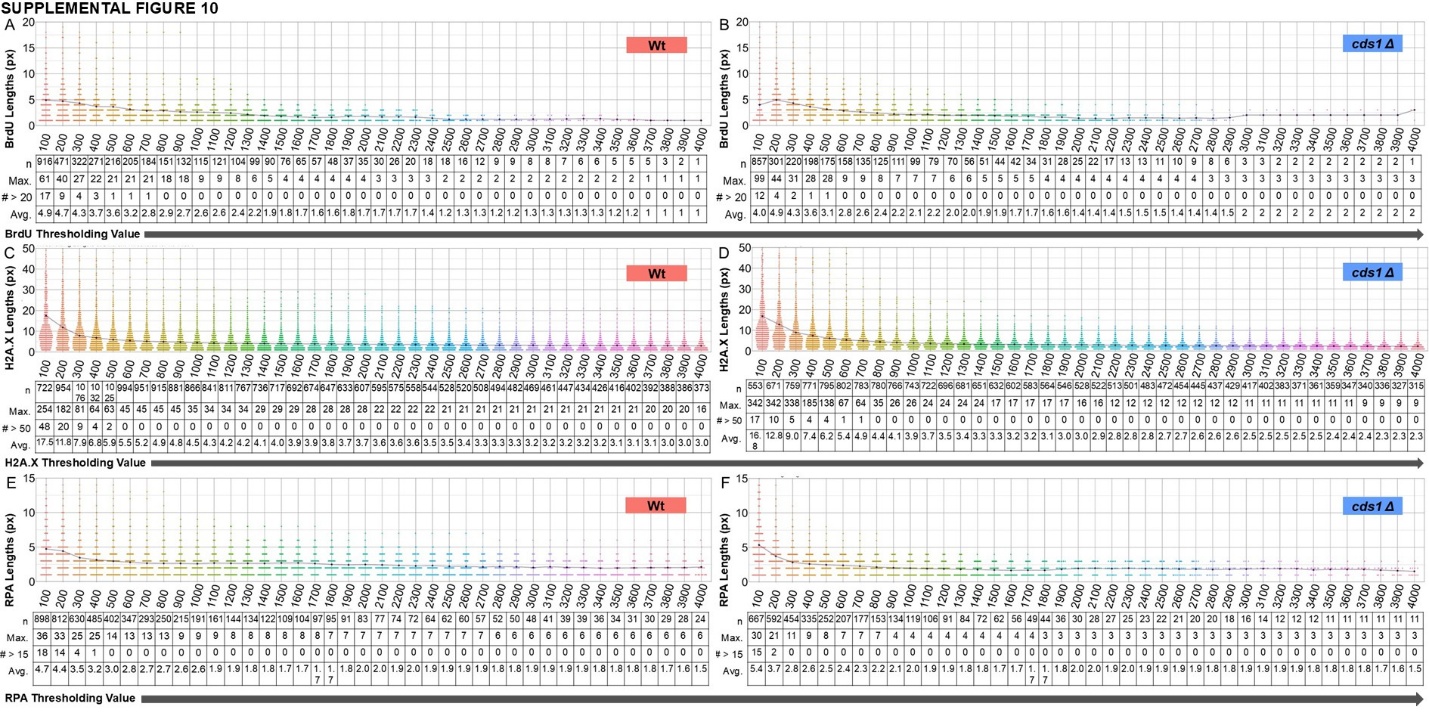


**Supplementary Figure 10: Increased fluorescent threshold values decrease BrdU, H2A.X, and RPA lengths.** The intensities of the line-traced chromatin fiber data were processed using R-ODD-BLOBS using different thresholds for each channel. Thresholds ranging from 100 to 4000 were used to test the effect of higher thresholds on the tract length of DNA synthesis (BrdU) or protein (H2A.X, RPA). A larger range of thresholding was used as the background of the microscopy image was higher. Each channel was iterated separately. A baseline “placeholder” threshold was used for each channel not tested. Baseline thresholds were calculated from a scatter plot of intensities (Supplementary Figure S1): 100 for DNA, 100 for BrdU, 200 for H2A.X, and 200 for RPA. Note that the chart scale best represents the patterns in each strain; outliers are indicated by the maximum values in the chart below each threshold bin. (A-B) Bee swarm plots of the tract lengths in BrdU in wt, and *cds1Δ* at thresholds from 100- 4000. (C-D) Bee swarm plots of the protein lengths in H2A.X in wt, and *cds1Δ* at thresholds from 100- 4000. (E-F) Bee swarm plots of the protein lengths in RPA in wt, and *cds1Δ* at thresholds from 100- 4000.

**
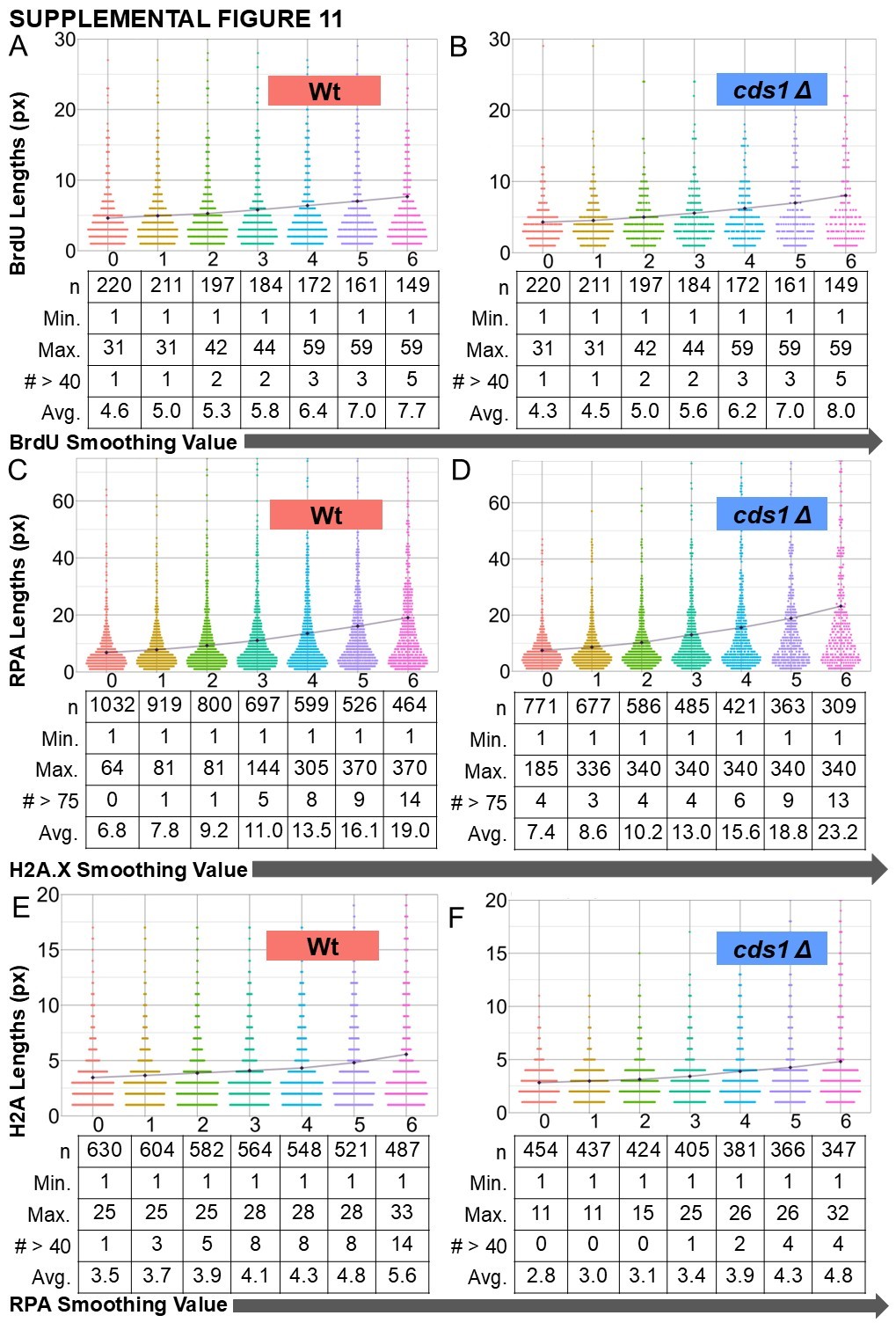
**

**Supplementary Figure 11: Larger smoothing values contribute to longer tract lengths for all of BrdU, H2A.X, and RPA.** The intensity of the line-traced chromatin fiber data was processed using R-ODD-BLOBS using previously determined threshold values: 100 for DNA; 300 for BrdU, RPA, and 400 for H2A.X. Smoothing links tracts together if the number of pixels is equal to or less than the “smoothing value” used. (A-B) Bee swarm plots of the tract lengths in BrdU in wt, and *cds1Δ* at smoothing values 0 px to 6 px. (C-D) Bee swarm plots of the tract lengths in H2A.X in wt, and *cds1Δ* at smoothing values 0 px to 6 px. (E-F) Bee swarm plots of the tract lengths in RPA in wt, and *cds1Δ* at smoothing values 0 px to 6 px.


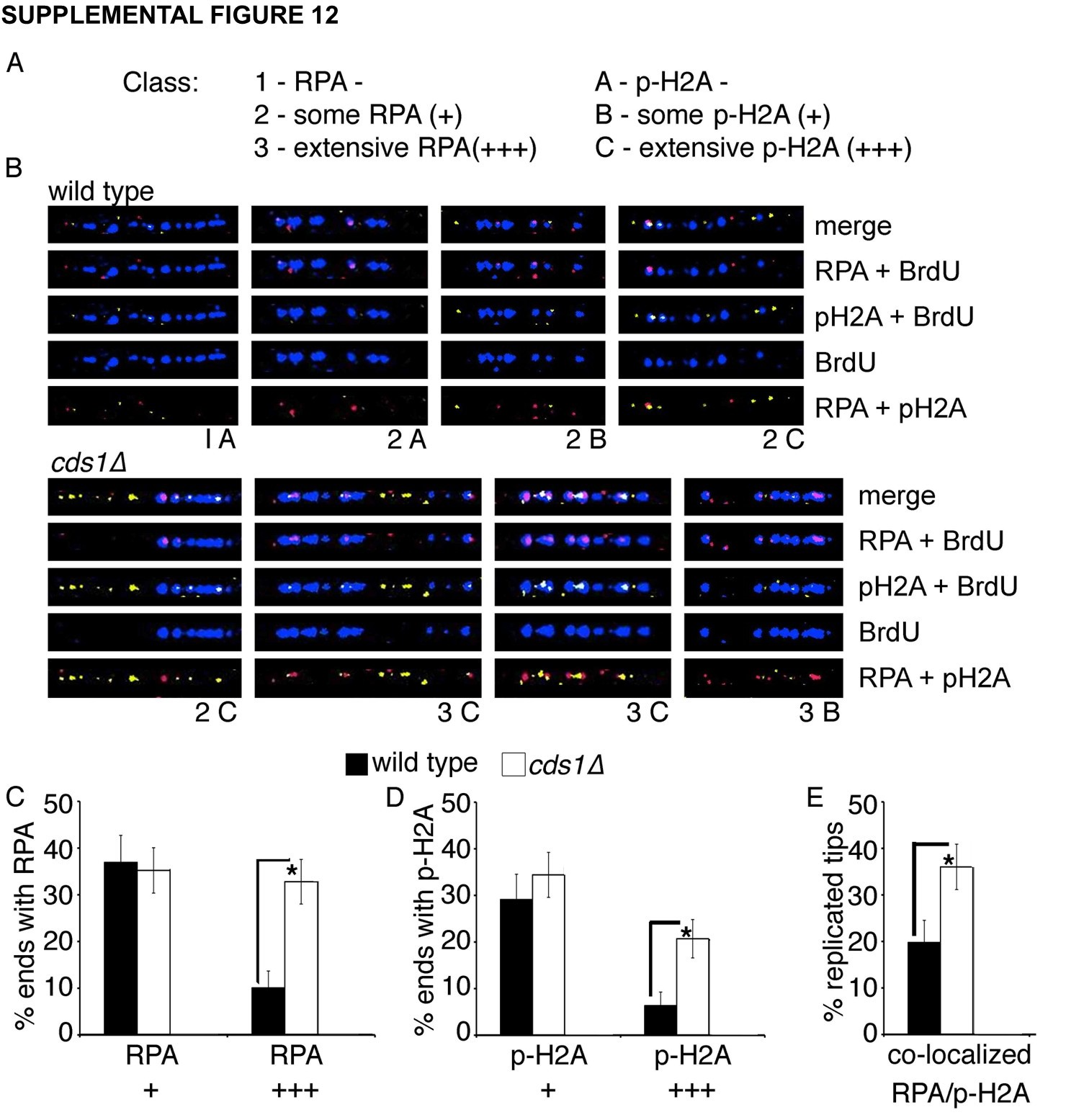


**Supplementary Figure 12: Visual inspection agrees that RPA and H2A.X accumulate at replicated tips after release.** Images of wt and *cds1Δ* cells blocked in HU (4 h) were released with BrdU and DNA fibers prepared 0.5h post-release. BrdU incorporation defines replicated zones. Replicated tracts were assessed visually using ImageJ to describe patterns of RPA and H2A.X and proximity to BrdU tract tips. The end of replicated tracts (tips, or potential fork zones) was defined as within 0.5 µm of the replicated tract tip (approximately 6 px in this microscope configuration) and was checked using ImageJ distance tool. The amount of RPA and H2A.X was scored manually, determined by spread of protein *(i.e.* length on fiber) and brightness (weak vs bright foci). Brightness was assessed using a threshold in ImageJ set at 2x above the background fluorescent intensity for each RPA or H2A.X channel (as in [23]).

(A) When quantifying spread fibers by eye, RPA or H2A.X protein signal intensity at BrdU can be classified as little/no signal (class 1 or A), moderate (+, class 2 or B), or extensive (+++, class 3 or C). Note that R-ODD-BLOBS was not used in this analysis.

(B) Examples of fibers around BrdU replicated regions for merged signals. Replicated tracts and proteins were compared; RPA and BrdU, H2A.X and BrdU, BrdU only, or RPA and H2A.X. RPA and H2A.X amount at BrdU-tips was classified as above. DNA signal (DAPI) was omitted for clarity. Scale bar 5 µm.

(C) Moderate RPA signal (RPA +) is found at BrdU-tips in both genotypes, but extensive (RPA +++) is significantly increased on *cds1∆* (* p<0.001).

(D) Extensive H2A.X (+++) is significantly increased at *cds1∆* replicated tips (* p<0.001).

(E) Direct RPA and H2A.X co-localization is greater at BrdU-tips in *cds1∆* (* p<0.001).


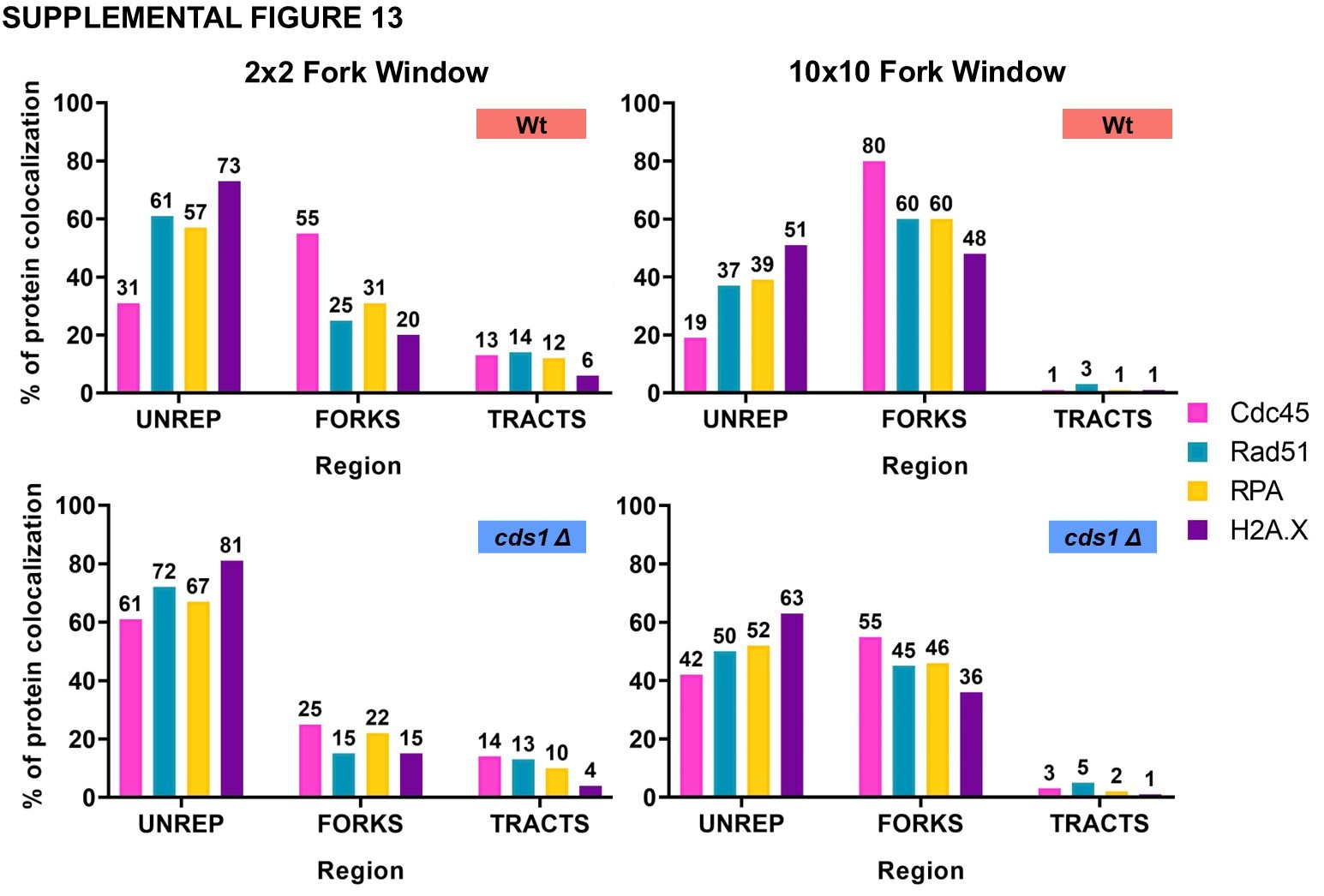


**Supplementary Figure 13: Fork-tip window analysis between wt and *cds1∆* highlights shift in region colocalization.** Data from figures 5D/E and 7D/E are presented together to compare how the proteins Cdc45, Rad51, H2A.X, and RPA interact with a 2x2 window or a 10x10 window around replicated tract tips.
